# Bioprospecting of *Aspergillus* sp. as a promising repository for anti-cancer agents: a comprehensive bibliometric investigation

**DOI:** 10.3389/fmicb.2024.1379602

**Published:** 2024-05-15

**Authors:** Himanshu Jangid, Sonu Garg, Piyush Kashyap, Arun Karnwal, Amrullah Shidiki, Gaurav Kumar

**Affiliations:** ^1^School of Bioengineering and Biosciences, Lovely Professional University, Jalandhar, Punjab, India; ^2^Department of Biotechnology, Mahatma Jyoti Rao Phoole University, Jaipur, Rajasthan, India; ^3^School of Agriculture, Lovely Professional University, Jalandhar, Punjab, India; ^4^Department of Microbiology, National Medical College & Teaching Hospital, Birgunj, Nepal

**Keywords:** *Aspergillus* species, anti-cancer agents, bioprospecting, natural products, bibliometric analysis

## Abstract

Cancer remains a significant global health challenge, claiming nearly 10 million lives in 2020 according to the World Health Organization. In the quest for novel treatments, fungi, especially *Aspergillus* species, have emerged as a valuable source of bioactive compounds with promising anticancer properties. This study conducts a comprehensive bibliometric analysis to map the research landscape of *Aspergillus* in oncology, examining publications from 1982 to the present. We observed a marked increase in research activity starting in 2000, with a notable peak from 2005 onwards. The analysis identifies key contributors, including Mohamed GG, who has authored 15 papers with 322 citations, and El-Sayed Asa, with 14 papers and 264 citations. Leading countries in this research field include India, Egypt, and China, with King Saud University and Cairo University as the leading institutions. Prominent research themes identified are “endophyte,” “green synthesis,” “antimicrobial,” “anti-cancer,” and “biological activities,” indicating a shift towards environmentally sustainable drug development. Our findings highlight the considerable potential of *Aspergillus* for developing new anticancer therapies and underscore the necessity for further research to harness these natural compounds for clinical use.

## 1 Introduction

Cancer, often described as a modern-day scourge, is not a singular disease but rather a complex constellation of disorders that share a common hallmark: the uncontrolled proliferation of cells. This unregulated growth often leads to the formation of tumors, which can invade adjacent tissues and spread to other parts of the body, a process known as metastasis (Baskar et al., 2014). The multifaceted nature of cancer, with its myriad types and subtypes, each with its unique genetic and environmental triggers, makes it a challenging disease to understand, diagnose, and treat. In the year 2018, around 18.1 million new instances of cancer and 9.6 million fatalities occurred on a global scale, underscoring the significant influence of cancer on global public health (Sung et al., 2021) The rates of cancer incidence and mortality have displayed fluctuations over time. For instance, in 2020, there were approximately 19.3 million fresh cancer cases and nearly 10.0 million deaths, with female breast cancer emerging as the most frequently identified form of cancer (Ferlay et al., 2021). Further Cancer statistics reveal that in 2022, approximately 1,918,030 new cancer cases and 609,360 deaths were projected, reflecting a continued decline in mortality due to advances in treatment, particularly for lung cancer (Siegel et al., 2023). In 2023, it is estimated that there will be 1,958,310 new cases and 609,820 deaths, with significant declines in cervical cancer incidence among younger women attributed to the HPV vaccine, showcasing the impact of preventive healthcare (Siegel et al., 2023).

These figures are not just numbers; they represent individuals, families, and communities affected by the disease, emphasizing the urgency to address this global health crisis. Over the decades, the scientific community has made remarkable strides in the field of oncology (Kruk et al., 2018). From the advent of chemotherapy, and targeted therapies, to the recent breakthroughs in immunotherapy, the landscape of cancer treatment has transformed dramatically. Patients today have access to a broader spectrum of therapeutic options than ever before, leading to improved survival rates and quality of life for many cancer types. Many current therapeutic regimens come with a host of side effects, some of which can be severe and diminish the quality of life (Meng et al., 2022). Resistance to treatment, either intrinsic or developed over time, is another significant hurdle. Furthermore, for some aggressive and rare cancers, effective treatments remain elusive. This backdrop underscores the continuous quest for novel anti-cancer agents. The ideal therapeutic would not only be effective in halting the progression of the disease but would also exhibit minimal toxicity, ensuring better patient compliance and outcomes (Biswas et al., 2019). The exploration for such agents is expansive, spanning from synthetic compounds to natural products, with the latter holding significant promise due to their vast structural diversity and proven track record in drug discovery. While significant advancements have been made in the realm of cancer therapeutics, the quest for novel, effective, and less toxic anti-cancer agents continues (Vora et al., 2023). Natural substances are essential in the field of cancer treatment, playing a crucial role in influencing various processes like cellular proliferation, differentiation, apoptosis, angiogenesis, and metastasis. These mechanisms are vital for treating diverse cancer types, as highlighted by Singh et al. (2023). Furthermore, these natural products exhibit the ability to regulate autophagy, providing novel possibilities for the development of mechanism-oriented anticancer medications by modulating this pivotal cellular homeostasis process (Al-Bari et al., 2021).

In the vast realm of natural products, fungi have emerged as a prolific source of bioactive compounds with therapeutic potential. Among fungi, the genus *Aspergillus* holds a special place in bioprospecting endeavors. *Aspergillus* species have been recognized for their ability to produce a myriad of secondary metabolites, many of which exhibit potent biological activities (Bok et al., 2006). These metabolites range from antibiotics, and immunosuppressants, to agents with anti-cancer properties. *Aspergillus* species produce a diverse array of compounds, such as alkaloids, butenolides, terpenoids, and polyketides, known for their anti-cancer activities. These secondary metabolites exhibit promising anti-cancer activity, with variable toxic potential and apoptotic potential, suggesting their potential use as supplementary agents in existing anti-cancer drug regimens (Siddhardha et al., 2010). Further, specific strains like *Aspergillus* sp. strain F1544 have been identified to produce compounds with noteworthy antileishmanial and moderate anticancer activities (Martínez-Luis et al., 2012). Bibliometric analysis of the research landscape in this field reveals a significant body of work focusing on various anticancer compounds produced by *Aspergillus* species. This analysis highlights the importance of genetic research in understanding the biosynthetic pathways of these metabolites. For instance, studies have provided comprehensive annotations of secondary metabolite biosynthetic genes and gene clusters in *Aspergillus* species, aiding in the discovery of novel secondary metabolites and potential anti-cancer drugs (Inglis et al., 2013). Overall, the exploration of *Aspergillus* species in the search for anti-cancer agents, supported by a robust bibliometric analysis, represents a generative field of study. The Potential of bioactive compounds offers promising avenues for the development of new therapeutic strategies in oncology, highlighting the potential of this genus in bioprospecting endeavors.

## 2 Literature review

### 2.1 *Aspergillus* species diversity and bioprospecting potential

*Aspergillus* sp. a genus consisting of several hundred mold species, is found in various climates worldwide. This genus was first identified in 1729 by Pier Antonio Micheli. It includes notable species like *A. flavus*, a plant pathogen and common cause of aspergillosis; *A. fumigatus*, prevalent in immunocompromised individuals; *A. nidulans*, used in cell biology research; *Aspergillus niger*, in the chemical industry; and *A. oryzae* and *Aspergillus sojae*, used in East Asian cuisine (Yadav et al., 2019). The genus is divided into six subgenera, further split into 27 sections. *Aspergillus* species are primarily conidial fungi, often in an asexual state, but some have a sexual state in Ascomycota. They thrive in high osmotic pressure environments and are highly aerobic, found in oxygen-rich settings, and grow on carbon-rich substrates like monosaccharides and polysaccharides. This makes them common contaminants of starchy foods and plants (de Vries et al., 2017).

Ecologically, *Aspergillus* sp. species are significant mycotoxin producers. Their presence in ecosystems is controlled by factors such as microclimate, substrate availability, and water activity. They play crucial roles as decomposers of organic materials and have a substantial impact on ecosystems, agriculture, food production, biotechnology, and human health. The effects of climate change might lead to the emergence of new *Aspergillus* sp. species and increased mycotoxin contamination risks, as these species can adapt to nutritional and biophysical challenges (Navale et al., 2021). With most of their gene clusters remaining silent, they are a potential source of underexplored bioactive compounds. In summary, *Aspergillus* sp. is important in both scientific research and industry, as well as in ecological contexts, due to its roles in decomposition, mycotoxin production, and its impact on agriculture and food safety (Nji et al., 2023). The complexity of its interactions in various environments and the potential for discovering new bioactive compounds from its species underscore the importance of continued research in this area. One additional aspect to consider is the health implications of *Aspergillus* sp. While certain species are beneficial for industrial and culinary uses, others can pose health risks, particularly for people with weakened immune systems (Vaou et al., 2021). For example, *Aspergillus fumigatus* is a common cause of aspergillosis, an infection that can be severe in immunocompromised individuals. Apart from *Aspergillus fumigatus*, a frequent cause of aspergillosis in immunocompromised individuals, *Aspergillus terreus* is also clinically important. This species is associated with several severe conditions such as allergic bronchopulmonary aspergillosis, *Aspergillus* bronchitis, and invasive aspergillosis, underscoring the importance of understanding and researching the pathogenic capabilities of different *Aspergillus* species.

Moreover, the production of mycotoxins by some *Aspergillus* species, notably *A. flavus* and *A. parasiticus* which produces aflatoxins, is a significant concern in food safety (Latgé, 1999). Aflatoxins are potent carcinogens and pose a serious risk to both human and animal health, contaminating crops like grains, nuts, and spices. It’s also worth noting the adaptability and resilience of *Aspergillus* sp. (Dhakal et al., 2023). Their ability to thrive in diverse and often harsh environments, such as high-sugar or high-salt conditions, highlights their evolutionary success. This resilience, however, also means that they can readily colonize and contaminate food and environments, posing ongoing challenges in food storage, agricultural practices, and indoor air quality. In conclusion, while *Aspergillus* sp. are ecologically and industrially important, their impact on human health and agriculture through disease and mycotoxin production is a critical area of ongoing research and monitoring (Dhakal et al., 2023).

### 2.2 Genetic makeup of *Aspergillus* contributes to its potential in drug discovery

The genetic composition of *Aspergillus*, a group of filamentous fungi, is of great significance in drug discovery due to its capacity to produce a diverse array of secondary metabolites (SMs). These metabolites, such as mycotoxins, polyketides, and peptides, are well-known for their varied biological activities, making them promising candidates for pharmaceutical research and development. *Aspergillus* species can synthesize over 200 different secondary metabolites thanks to their intricate genetic structure, which includes unique enzymatic pathways. These pathways play a vital role in the fungus’s survival and adaptation to different environments, while also offering a valuable source of bioactive compounds for drug discovery (Keller, 2019). Therefore, comprehending how metabolite production is genetically controlled in *Aspergillus* is crucial for leveraging its potential in creating innovative therapeutics.

#### 2.2.1 Genome mining and secondary metabolites

Recent advancements in whole genome sequencing methods have greatly improved our comprehension of *Aspergillus* species, revealing a diverse genetic reservoir poised for the production of secondary metabolites (SMs). Detailed genetic investigations have identified numerous gene clusters responsible for SM biosynthesis, many of which remain inactive or express at low levels under standard laboratory conditions (Romsdahl and Wang, 2019). This discovery suggests a potential treasure trove of new bioactive compounds suitable for pharmaceutical purposes. To unlock this potential, researchers have employed innovative techniques such as modifying growth conditions, using chemical triggers, and employing gene editing tools like CRISPR-Cas9 to activate these dormant gene clusters. For instance, specific adjustments to nutrient levels in growth media have induced the expression of previously inactive biosynthetic pathways, creating novel SMs (Dzobo, 2022).

Additionally, the use of bioinformatics tools has enabled scientists to predict the structure of new compounds by analyzing the genetic sequences responsible for their formation. This method has facilitated the identification of promising drug candidates even before chemical synthesis, significantly expediting the discovery process (Bayat, 2002). One notable breakthrough involved activating a previously unidentified gene cluster in *Aspergillus nidulans,* leading to the discovery of nidulanins A and B, compounds potentially possessing anti-inflammatory properties. This was achieved by overexpressing specific transcription factors that regulate SM biosynthesis, showcasing how genetic insights can guide the exploration and exploitation of fungal secondary metabolites (Wang et al., 2021).

These initiatives in genome exploration and genetic manipulation not only deepen our understanding of fungal biology but also have the potential to transform drug discovery by presenting new blueprints for therapeutic agents. Through these comprehensive genomic investigations, *Aspergillus* remains a significant source of pharmacologically active compounds, aiding in the development of next-generation medications.

#### 2.2.2 Biosynthetic gene clusters

The magnitude of BGCs in a single filamentous fungal genome suggests that the secondary metabolite wealth of filamentous fungi like *Aspergillus* is largely untapped. BGCs are chromosomal architectures encoding necessary synthetases and/or synthases in the fungal genome, controlling the production of fungal secondary metabolites (Clevenger et al., 2017). Genome sequencing has shown diverse chemical applications of these metabolites. For instance, *Aspergillus ficuum,* a member of the *Niger* group of fungi, has been documented for its large number of secondary metabolites and mycotoxins, including ochratoxins, fumonisins, naphtha-pyrones, bicoumarins, malformins, asperazines, and alkaloids (Nielsen et al., 2009). These compounds have various applications in pharmaceuticals, cosmetics, and other industries. Further Table 1 details the diversity of secondary metabolites produced by different species of *Aspergillus* and other fungi, highlighting the associated BGCs, their applications, and strategies employed for activating these gene clusters. The information underscores the immense potential of these organisms in the biotechnology and pharmaceutical industries.

**Table 1:** Overview of biosynthetic gene clusters (BGCs) in *Aspergillus* species.

Fungal species	Secondary metabolites identified	Key applications	Regulatory gene involved	BGC activation strategies	Reference
*Aspergillus nigri*	Polyketides, Non-ribosomal Peptides, Terpenoids, etc.	Pharmaceuticals, Cosmetics	Genes controlling polyketide synthases and peptide synthetases	Using bioinformatics tools like antiSMASH	Wang et al. (2022)
*Aspergillus nidulans*	Lovastatin, Acetylaranotin, Butyrolactones, Territram, etc.	Cholesterol-lowering, Bioactivities	LaeA, a global regulator of secondary metabolism	CRISPR activation, heterologous expression	Guo and Wang (2014)
*Aspergillus ustus*	Sterigmatocystin, Viridicatumtoxin, etc.	Medicine, Industrial	AflR and AflS, regulators of mycotoxin production	Comparative genomics	Pi et al. (2015)
*Aspergillus fumigatus*	Antibiotics, Potential anti-cancer drugs	Pharmaceuticals	GliZ, a regulator of gliotoxin biosynthesis	Epigenetic manipulation to activate silent gene clusters	Inglis et al. (2013)
*Aspergillus terreus*	Lovastatin, Acetylaranotin, Butyrolactones	Pharmaceuticals, Bioactivities	LovE, regulator of lovastatin production	Advanced genome mining techniques	Guo and Wang (2014)
*Aspergillus oryzae*	Kojic acid, other polyketides	Food industry, Pharmaceuticals	AflJ, involved in kojic acid regulation	Co-cultivation techniques to mimic natural conditions	Brakhage and Schroeckh (2011)
*Aspergillus flavus*	Aflatoxins, other mycotoxins	Food safety	AflR, a key regulator of aflatoxin biosynthesis	Gene knockout and overexpression techniques	Uka et al. (2020)

#### 2.2.3 Pharmacological profiling

The pharmacological potential of *Aspergillus* species, such as *A. ficuum, has* been explored through techniques like Liquid Chromatography Quadruple Time-of-Flight Mass Spectrometry (LC-QToF-MS) and Gas Chromatography–Mass Spectrometry (GC–MS) (Shah et al., 2022). These studies focus on uncovering untargeted metabolites biosynthesized in liquid culture. Investigations include assessing the anti-inflammatory, acute oral toxicity, antibacterial, and free radical scavenging potentials of these fungal metabolites. Molecular docking analysis has also been used to understand how these mycocompounds interact with specific enzymes like DNA-polymerase of *Bacillus subtilis* and the inflammation-supporting enzyme cyclooxygenase-2 (Orozco-Cortés et al., 2023).

In summary, the genetic makeup of *Aspergillus* significantly contributes to its potential in drug discovery. Its genome harbors a plethora of biosynthetic genes that produce a diverse array of secondary metabolites, many of which have been identified as potential drug candidates. The ongoing exploration and understanding of these metabolites through advanced genomic and pharmacological techniques continue to open new avenues in the search for novel therapeutic agents.

### 2.3 Anti-cancer agents from *Aspergillus* species

*Aspergillus* species are emerging as promising candidates in the quest for innovative anti-cancer agents. Their distinctive metabolic capabilities allow for the synthesis of a wide range of bioactive compounds, demonstrating significant effectiveness against diverse cancer types (Kalimuthu et al., 2022). For example, optimizing the fermentation of *Aspergillus wentii* EN-48 resulted in a substantial increase in the yield of asperolide A, a potent agent against lung cancer (Teixeira et al., 2019). Similarly, *Aspergillus ochraceus* is recognized for producing nitrobenzoyl sesquiterpenoids with notable cytotoxicity against a variety of cancer cell lines, highlighting the adaptability of *Aspergillus* species in targeting different cancers (Fang et al., 2014).

**Figure 1:**
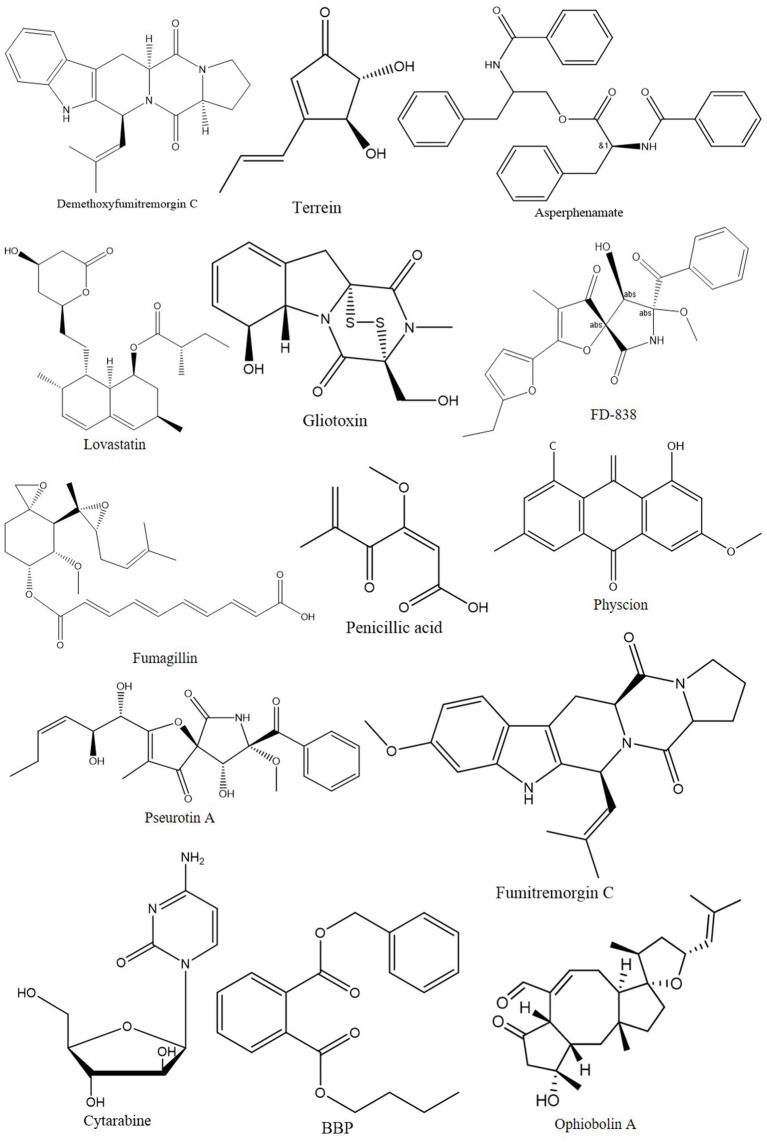
Chemical structure of anticancer compound reported from *Aspergillus* sp.

**Figure 2:**
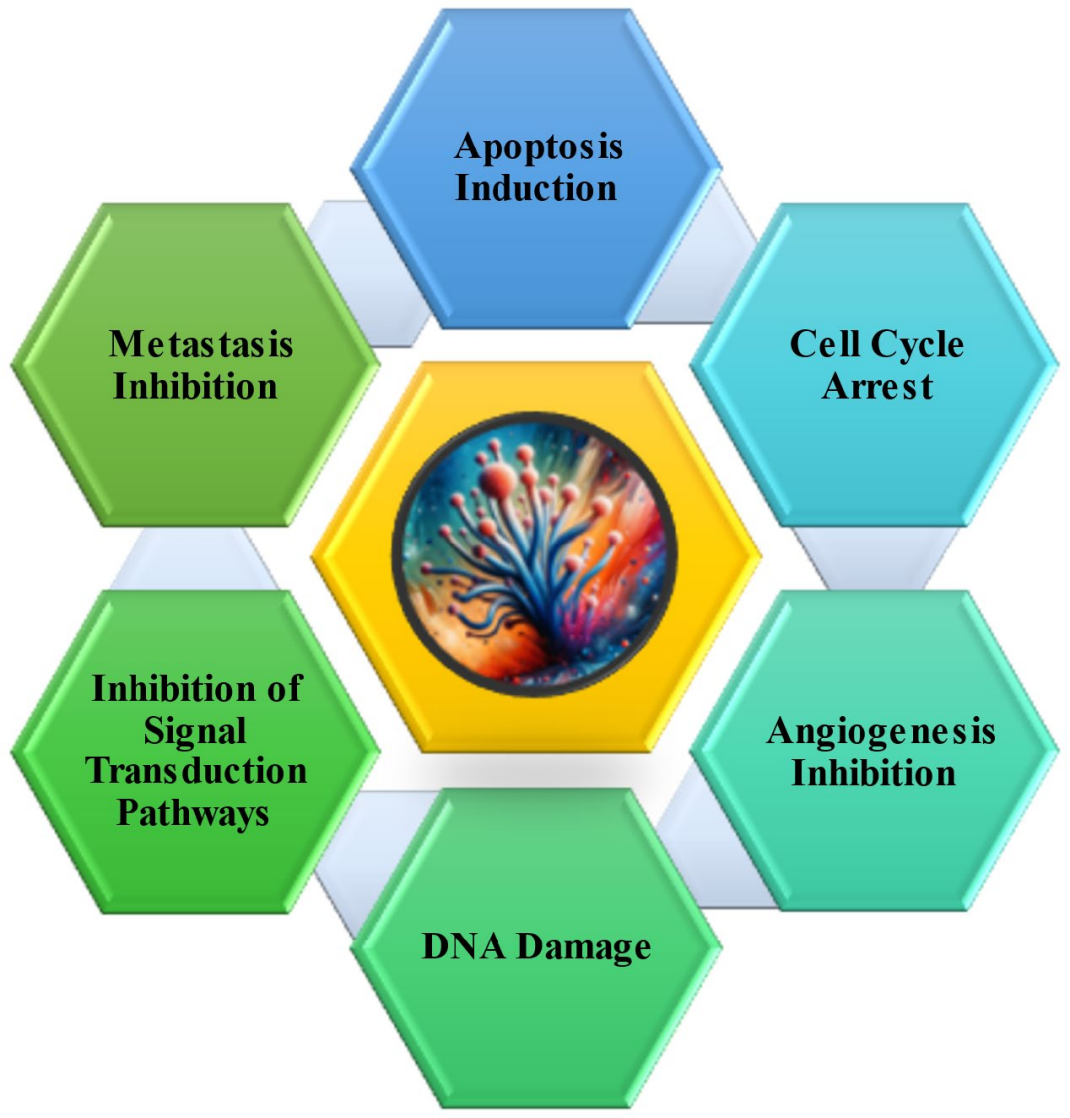
Various mode of action of anti-cancer agents produced from *Aspergillus* species (Adapted and modified from Bladt et al., 2013; Noman et al., 2021).

The anti-cancer properties of *Aspergillus* species are evident in their production of various compounds, including alkaloids, butenolides, terpenoids, and polyketides (Figure 1). This structural diversity contributes to their multifaceted approach to combating cancer (Bladt et al., 2013). Significantly, these species have been identified as prolific producers of anticancer compounds, effectively inhibiting cancer cell growth and inducing apoptosis (Figure 2), a crucial mechanism in cancer therapy (Greenwell and Rahman, 2015). Comparatively, while other fungi possess medicinal properties, the anti-cancer compounds of *Aspergillus* species appear to be more diverse and potent. For instance, *Trichoderma* species, known for their antifungal activities, lack the same breadth of anti-cancer potential as *Aspergillus* (Noman et al., 2021). Furthermore, *Aspergillus* species’ secondary metabolites, such as terpenes, alkaloids, and polyketides, exhibit greater cytotoxic potency against specific cancer cell lines, such as colorectal cancer, than those from many other natural sources. These findings suggest a heightened potential for *Aspergillus* species in the development of new cancer treatments (Shah et al., 2023). Additionally, these metabolites show promise as supplementary agents in existing anti-cancer drug regimens, potentially enhancing the efficacy of current treatments (Chehelgerdi et al., 2023).

The anti-cancer potential of *Aspergillus* is further emphasized by its ability to produce a diverse array of bioactive metabolites, ranging from potential anti-cancer drugs to therapeutic antibiotics. This highlights the versatility and broad applicability of compounds derived from *Aspergillus,* positioning it as a significant and versatile contributor to drug discovery, particularly in the field of cancer research (Dehelean et al., 2021). Subsequent Table 2 outlines some of these key classes of anti-cancer compounds along with their mechanism of action and known targets produced by *Aspergillus* species.

**Table 2:** Various anti-cancer compounds reported from *Aspergillus* sp. and their mechanism of action.

*Aspergillus* species	Specific compounds	Class of compound	Mechanism of action	Cancer type	IC 50	Reference
*A. terreus*	Lovastatin	Polyketides	HMG-CoA reductase inhibitor	Cervical cancer	2.3 nmol/L in rat liver cells and 5 nmol/L in the human liver hepatocellular carcinoma cell line	Jamil (2015)
*A. fumigatus*	Fumagillin	Alkaloids	Methionine aminopeptidase 2 inhibitor	Liver Cancer cell	10 ± 3.1 μg/mL against HeLa cell lines	Zhang et al. (2023)
*Aspergillus* sp. *FT1307*	Aspochalasin H1	Polyketides	Inhibits angiogenesis by downregulating VEGF, p-p38, p-ERK, p-VEGFR-2, and p-Akt signaling pathways	Breast and ovarian cancer cell lines	17.29 μM against ovarian cancer cell line	Zhu et al. (2011)
*Aspergillus fumigatus*	Demethoxyfumitremorgin C	Polyketides	Inhibits the proliferation of PC3	Prostate cancer	Human pancreatic cancer cells including AsPC-1, SW1990, and PANC-1 cells, with values ranging from 1.2 to 15.6 μM	Kim et al. (2017)
*Aspergillus terreus JAS-2*	Terrein	Polyketides	Inducing apoptosis	Lung cancer cell line A-549	121.9 ± 4.821 μg/mL against the human lung cancer cell line A-549	Goutam et al. (2017) and Porameesanaporn et al. (2013)
*Aspergillus wentii EN-48*	Asperolide A	Terpenoids	Cell cycle arrest, Apoptosis, and Osteoclast formation.	Lung cancer	1.53 ± 0.17 μM against hepatoma cells	Lv et al. (2023)
*Aspergillus glaucus*	Aspergiolide A	Anthraquinones	Cell cycle, inducing apoptosis, and inhibiting ERK activation.	Cervical cancer and Chronic myelogenous leukemia cells	8.9, 7.8, and 18.4 μM against A375, SW-620, and HeLa carcinoma cell lines	Yuan et al. (2017)
*Marine-derived unidentified Aspergillus species*	Physcion and 2-(2′,3-epoxy-1′,3′,5′-heptatrienyl)-6-hydroxy-5-(3-methyl-2-butenyl) benzaldehyde	Benzaldehyde	Target cancer cells in nutrient-deficient conditions, inhibiting mitochondrial ETC	Breast adenocarcinoma cell line MCF-7	6.0 and 1.7 μM against human pancreatic carcinoma PANC-1 cells under glucose-deficient conditions	Abdel-Naime et al. (2020)
*Marine algae-derived Aspergillus fungi*	Psychrophilin E	Cyclotripeptides	Anti-proliferative activity against various human cancer cell lines	Lung, Breast, and Colon Cancer	0.58 μM, 1.0 μM and 28.5 μM against MCF-7, H460 and HCT116 cell line	Ebada et al. (2014)
*Marine-derived Aspergillus* sp.	Asperphenin A	Lipopeptidyl benzophenone	Inhibits colon cancer cell growth by triggering microtubule disassembly and apoptosis	Colon cancer cells and Breast cancer	0.84 μM against CRC cells and 6.48 μM against breast cancer cells	Bae et al. (2020)
*Marine-derived Aspergillus* sp.	Asperphenamate	Lipopeptidyl Benzophenones	Inhibition of proteasome activity	Lung Cancer and breast cancer	T47D: 92.3 μM MDA-MB-231: 96.5 μM HL-60: 97.9 μM	Martínez-Luis et al. (2012)
*Aspergillus* sp.	Gliotoxin	Polyketides	Induction of apoptosis and immunosuppression	Human cervical cancer and colorectal cancer cells	0.6 μg/mL towards HT-29 colorectal cancer cells.	Nguyen et al. (2013)
*Aspergillus* sp. *strain F1544*	Pseurotin A, 14-norpseurotin A, FD-838, pseurotin D, and fumoquinone B	Spirocyclic lactones.	Inhibits PCSK9 secretion and its interaction with LDL receptor, suppressing breast cancer progression.	Hormone-dependent breast cancer.	0.51 to 29.3 μM against glioma cells	Abdelwahed et al. (2020)

*Aspergillus* species are a rich source of diverse and biologically active secondary metabolites, encompassing polyketides, terpenoids, peptides, and alkaloids, each with distinct anti-cancer properties. Investigating these compounds through structure–activity relationships (SAR) provides crucial insights for future oncological treatments (Hu et al., 2020). Notably, polyketides like lovastatin from *Aspergillus terreus* and terpenoids such as Asperolide A exhibit significant anti-cancer potential. Lovastatin, a well-studied compound, exemplifies the importance of specific structural elements; its hydroxylated open-ring form inhibits HMG-CoA reductase, a key enzyme in the mevalonate pathway, disrupting tumor growth at a biochemical level (Srinivasan et al., 2022).

Terpenoids, known for their rigid structure and functional diversity, interact with cellular targets intricately. For instance, asperolide A induces apoptosis and cell cycle arrest, possibly by interacting with regulatory proteins. Functional groups like epoxides within terpenoids enhance molecular reactivity and target specificity, highlighting the connection between chemical structure and biological activity (Thoppil and Bishayee, 2011).

In developing anti-cancer agents from *Aspergillus*, advanced screening methods are crucial to identify compounds with novel structures and increased potency. Leveraging biosynthetic gene clusters (BGCs) through genomic tools can expedite the identification and modification of these metabolites, improving pharmacokinetics and therapeutic efficacy. Detailed mechanistic studies will refine these compounds’ applications against specific cancer types, enriching the pipeline of innovative therapies and maximizing the therapeutic potential of *Aspergillus*-derived compounds in oncology.

### 2.4 Bioprospecting approaches

#### 2.4.1 Isolating and characterizing *Aspergillus* strains

In the intricate field of mycology, sophisticated molecular techniques are employed for the isolation and characterization of *Aspergillus* strains, ensuring precise identification and detailed analysis. A key method involves ribosomal DNA (rDNA) analysis which is highly valuable due to the conserved nature of rDNA sequences across species, making it an excellent marker for fungal identification (Diba et al., 2014). Additionally, microsatellite Single Nucleotide Polymorphisms (SNPs) enable high-resolution genetic profiling, allowing researchers to differentiate closely related strains and assess genetic diversity within populations. Another important technique is Restriction Fragment Length Polymorphism (RFLP), which examines the pattern of DNA fragments produced by restriction enzymes (Cruz and Buttner, 2008). This method plays a crucial role in mapping genetic relationships and variations among *Aspergillus* strains, providing insights essential for both fundamental research and practical applications. Furthermore, Multilocus Sequence Typing (MLST) is used to gain a comprehensive view of the genetic landscape by analyzing multiple genetic loci, which is pivotal for understanding the evolutionary paths and relationships among different fungal strains (Uran-Velasquez et al., 2022).

For rapid detection and quantification of specific strains like *Aspergillus* flavus, real-time Quantitative PCR (qPCR) has been developed. This technique not only detects minute amounts of DNA but also provides quantitative data crucial for pathogen monitoring and management in clinical and agricultural settings (Mitema et al., 2019). Integrating these DNA-based techniques with physical methods such as Percoll density gradient centrifugation, which separates strains based on cell density, and isoenzyme analysis, which distinguishes strains by their metabolic enzyme profiles, offers a robust framework for comprehensive characterization of *Aspergillus* species (Huang et al., 2015).

Moreover, the use of universal fungal barcodes and functional gene analysis enhances species-level identification precision, consolidating the role of genetic tools in studying fungal diversity and pathogenicity in depth. Collectively, these methods deepen our understanding of genetic and phenotypic diversity within the *Aspergillus* genus, paving the way for innovative applications in biotechnology and medicine (Xu, 2016).

#### 2.4.2 Screening and identification of bioactive compounds

**Figure 3:**
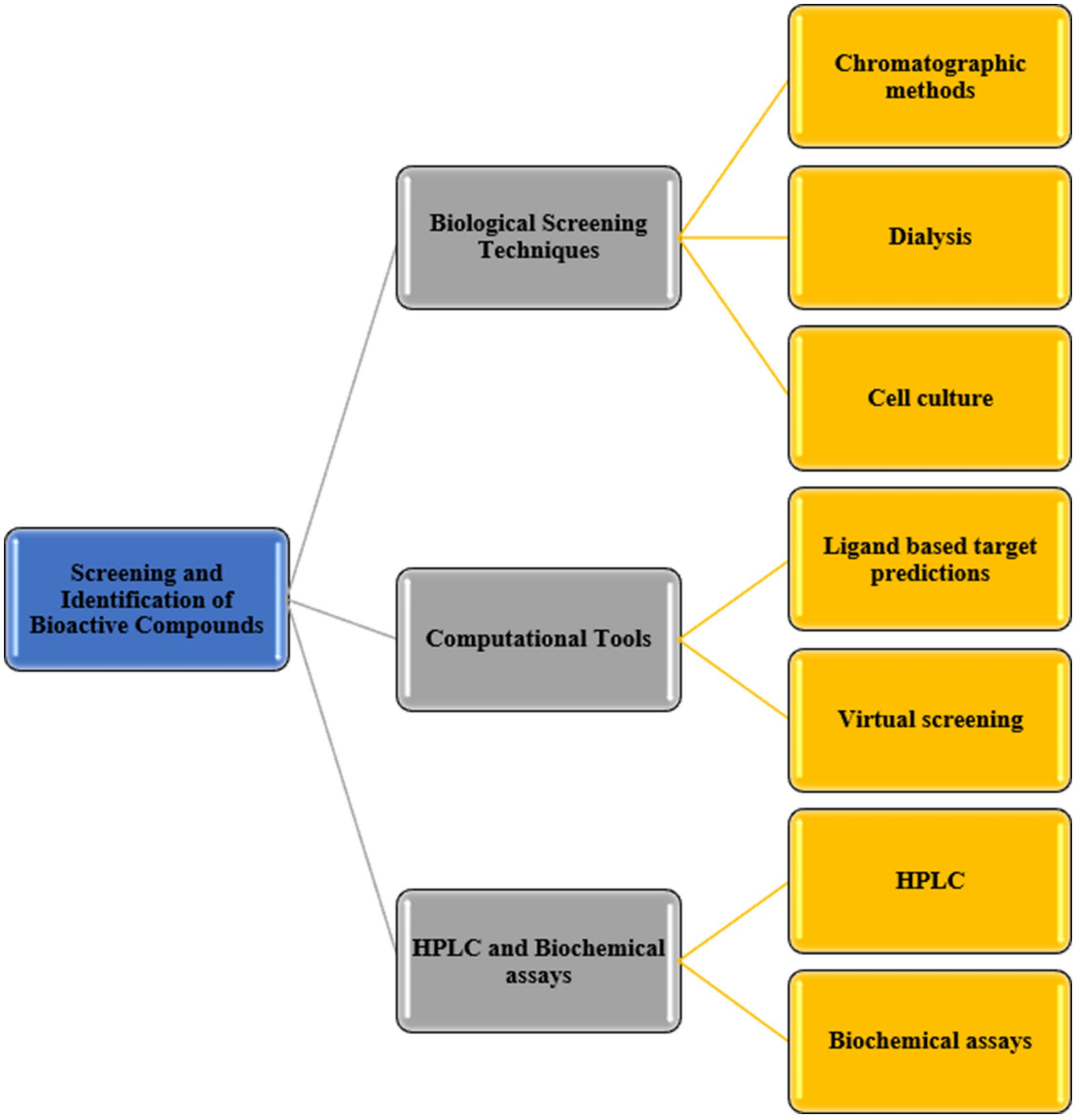
Detailed flow chart for the screening and identification of bioactive compounds adapted and modified from Zhang et al. (2023) and Yang et al. (2021).

Sophisticated approaches for the screening and recognition of bioactive compounds encompass a diverse set of methods. Crucial roles are played by biological screening techniques such as cell culture, dialysis, and various chromatographic methods (Fu et al., 2019). Computational tools are employed in virtual screening and ligand-based target prediction methods to identify potential compounds (Yang et al., 2021). Additionally, techniques like HPLC, when combined with biochemical assays, provide efficient screening capabilities without necessitating the purification of compounds (Zhang et al., 2023). A detailed Flow Chart for the Screening and Identification of Bioactive Compounds is illustrated in Figure 3.

#### 2.4.3 Integration of omics technologies

Significant progress has been made in the utilization of omics technologies in bioprospecting. Recent advancements involve the incorporation of genomics, transcriptomics, proteomics, and metabolomics, leading to improved biotechnological production of valuable natural products (Dai and Shen, 2022). Additionally, omics technologies are increasingly paired with machine learning and artificial intelligence to further enhance bioprospecting objectives (Cembrowska-Lech et al., 2023).

#### 2.4.4 Role of synthetic biology

Synthetic biology plays a pivotal role in advancing compound production and refinement. Progress in synthetic biology has resulted in the creation of microbial strains equipped with optimized biosynthetic pathways, enhancing the synthesis of value-added compounds (Chu et al., 2021). Additionally, synthetic biology tools aid in the exploration and optimization of gene clusters responsible for secondary metabolites, thereby significantly contributing to the development of new drugs derived from natural sources (Breitling and Takano, 2016). These comprehensive approaches and methodologies in bioprospecting offer a detailed understanding of the processes involved in isolating, characterizing, screening, and producing bioactive compounds from diverse biological sources. The amalgamation of advanced molecular techniques, omics technologies, and synthetic biology has markedly increased the efficiency and effectiveness of bioprospecting endeavors (Sekurova et al., 2019).

## 3 Methodology

### 3.1 Research design

This study employs a bibliometric analysis to comprehensively investigate the research landscape surrounding the bioprospecting of *Aspergillus* species for anti-cancer agents. Bibliometric analysis is a quantitative approach used to assess the impact and development of a field of research by analyzing patterns in academic literature (Xu et al., 2023).

### 3.2 Data sources

The data for this bibliometric study were extracted from Scopus databases. This database was chosen due to its extensive coverage of life sciences, and biomedical research, and its robust bibliometric data (Baas et al., 2020).

### 3.3 Search strategy

A systematic search was conducted using a combination of keywords and phrases related to “*Aspergillus* species,” “Bioprospecting,” and “Anti-cancer.” Boolean operators (AND, OR) were used to refine the search. The search was limited to articles published in English (Guo et al., 2023).

### 3.4 Inclusion and exclusion criteria

Articles were included if they were primary research articles, or meta-analyses focusing on the bioprospecting of *Aspergillus* species for anti-cancer agents. Excluded were non-English articles, Reviews, conference abstracts, books, book chapters, and articles unrelated to the specific focus of *Aspergillus* species as a source for anti-cancer compounds (Bezerra et al., 2021).

### 3.5 Data extraction

For each article, the following information was extracted: title, author(s), year of publication, journal name, number of citations, and keywords. The bibliometric criteria chosen for this analysis were carefully selected to illuminate the diverse landscape of *Aspergillus* research and its evolution over time. The title of each publication offers fundamental insights into the central themes and scope of the research, indicating shifts in scientific focus and emerging areas of interest. The names of the author(s) highlight key contributors, aiding in the mapping of expert networks and identifying pivotal figures whose work has significantly advanced the field. Analysis based on the year of publication reveals the historical trajectory of research efforts, pinpointing temporal trends and periods of breakthroughs in *Aspergillus*-related studies. The journal name where each study is published serves as an indicator of the research’s reach and relevance, reflecting the community’s engagement with and acknowledgment of the work. The number of citations received by a paper acts as a measure of its influence and the resonance of its findings within the broader scientific discourse. Lastly, the keywords used in the studies provide a vocabulary of the field’s focal points, revealing prevalent topics, methodological approaches, and conceptual linkages that characterize the body of *Aspergillus* literature. Together, these six criteria form a robust framework for bibliometric investigation, facilitating a comprehensive analysis that captures both the breadth and depth of the research domain (Kalantari et al., 2017).

### 3.6 Bibliometric analysis

- Publication Trend Analysis: To observe the growth or decline in research activity over time (Nelis et al., 2022).
- Citation Analysis: To assess the impact of the research field (Brown and Gardner, 1985).
- Co-occurrence Network Analysis: To identify the most frequent and influential keywords, indicating major research themes (Zhang et al., 2019).
- Co-authorship Analysis: To examine collaboration patterns among authors and institutions (Fonseca et al., 2016).
- Thematic Mapping: To analyze trends and research gaps (Parlina et al., 2020).

### 3.7 Tools for analysis

Bibliometric data were analyzed using tools such as Vosviewer for mapping & visualizing bibliometric networks. Biblioshiny, based on the R language script uses analysis of scientific production (Aria and Cuccurullo, 2017).

## 4 Results and discussion

**Figure 4:**
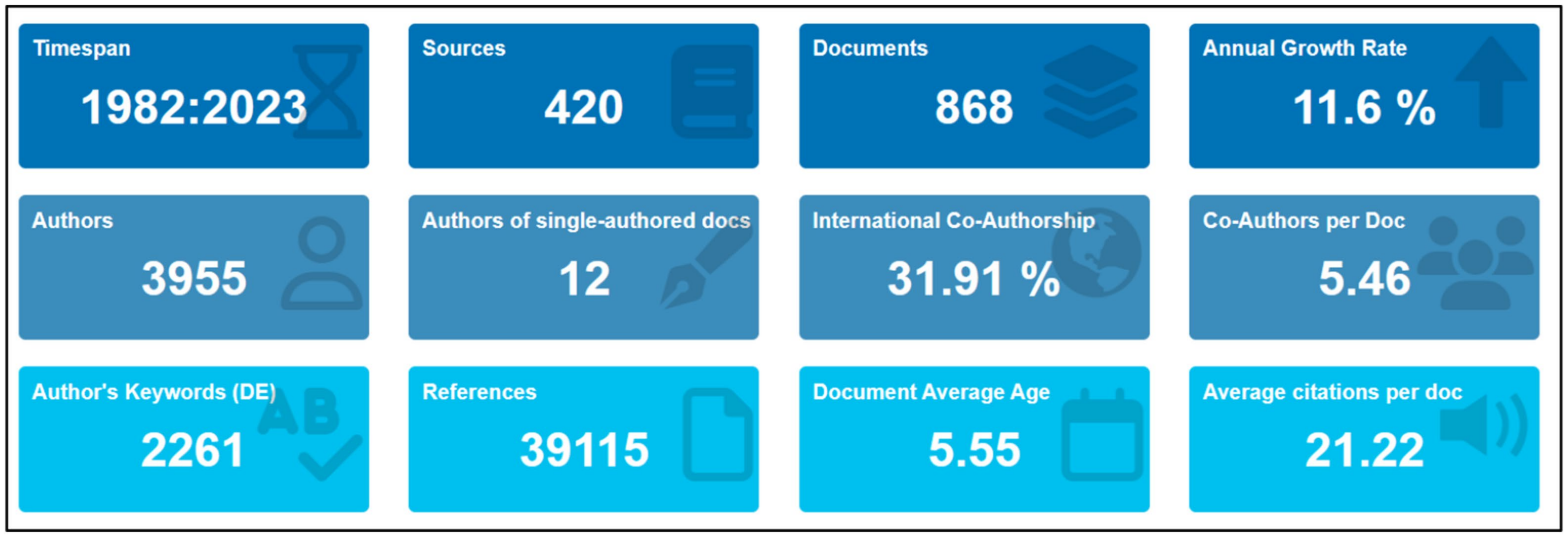
Overview of scientific data of anticancer studies on *Aspergillus* sp. collected from Scopus database on November 10, 2023.

Figure 4 offers a comprehensive overview of diverse bibliometric statistics related to the exploration of *Aspergillus species* as a promising reservoir for anti-cancer agents. Spanning from 1982 to 2023, the data encapsulates more than four decades of scholarly endeavors, encompassing 420 sources distributed across various journals, conference proceedings, and databases. The total of 868 documents, including research papers, reviews, and case studies, underscores the depth and breadth of exploration within this field. Demonstrating a robust and escalating interest, the field exhibits an annual growth rate in publications of 11.6%. The collaborative nature of this research is emphasized by the involvement of 3,955 authors in contributing to the body of work, with 12 instances of single-authored documents suggesting a prevalent collaborative norm. Noteworthy is the international co-authorship present in 31.91% of the documents, accentuating the global collaborative efforts, while an average of 5.46 co-authors per document further underscores the cooperative essence of the research community. The research’s diverse focus areas are evident in the 2,261 author’s keywords, reflecting a multifaceted exploration of *Aspergillus species.* With a total of 39,115 references cited, the research foundation is extensive, actively contributing to scholarly dialogue. The average document age of 5.55 years implies a recent emphasis or sustained relevance of older works, complemented by an impressive average of 21.22 citations per document, highlighting the research’s impact and significance in this field. In summary, this infographic provides a detailed glimpse into the dynamic landscape of *Aspergillus*-related anti-cancer research, revealing a substantial and expanding body of work. The collaborative, globally engaged, and impactful nature of the research community is apparent, affirming its considerable influence on the scientific literature.

### 4.1 Annual scientific production

**Figure 5:**
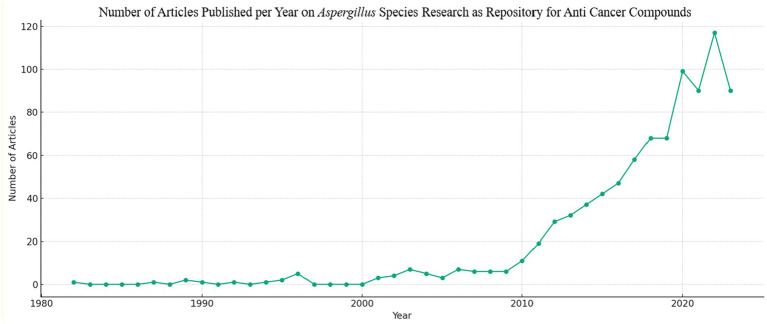
Annual publication data of scientific papers reported the anticancer studies on *Aspergillus* sp. collected from Scopus Database (between 1982 and 2023).

Figure 5 is a line graph depicting the progression of annual publications on *Aspergillus* species as potential sources of anti-cancer agents. An analysis based on the graph’s elements is as follows:

- X-axis (Publications Year): This axis delineates a timeline from 1982 to 2022, indicating the years in which publications were released.
- Y-axis (Number of Publications): The vertical axis denotes the quantity of publications, ranging from 0 to 140.
- Trend Line: Represented by a blue line, it illustrates the annual count of publications, reflecting the research output over time.

Observations from the graph include:

1. Gradual Increase: The number of publications exhibits a gradual rise over time, marked by intermittent fluctuations. This implies a growing scientific interest and exploration of *Aspergillus* species for their anti-cancer potential.
2. Uptrend Post-2000: Notably, an upward trend becomes apparent around the year 2000, with more pronounced growth post-2010.
3. Peak in 2020–2022: The highest concentration of publications occurs between 2020 and 2022, indicating a recent surge in research activity.

This graph effectively illustrates the expanding body of research into the medicinal properties of *Aspergillus.* It signifies the increased prominence of scientific exploration in this domain over the last two decades. Factors contributing to the rise in publications may include advancements in research methodologies, augmented funding, an influx of researchers into the field, or a combination of these influences.

*Aspergillus* species are recognized for producing diverse secondary metabolites, some possessing anti-cancer properties. The depicted trend underscores the significance of this genus in the quest for novel anti-cancer compounds, with the potential to advance drug and therapy development. Furthermore, it highlights the heightened awareness within the scientific community regarding the potential of natural products in drug discovery, emphasizing the ongoing necessity to explore and assess such biological resources (Kousar et al., 2022).

### 4.2 Publication distribution based regions

**Figure 6:**
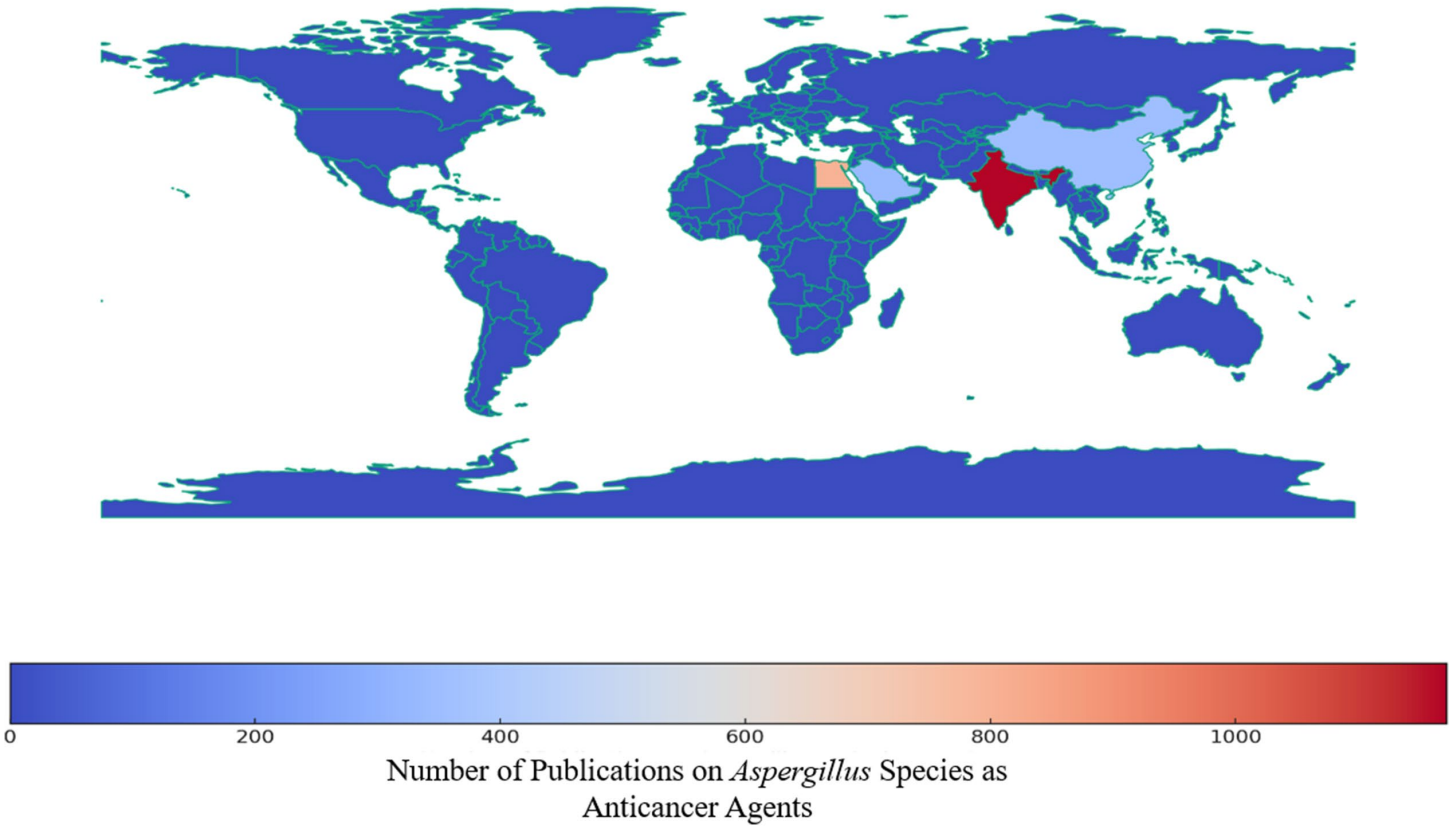
Countries scientific publication frequency of scientific papers reported the anticancer studies on *Aspergillus* sp. collected from Scopus database (between 1982 and 2023).

Figure 6 shows the global distribution of scientific publications related to *Aspergillus* species as a source of anticancer agents. The color intensity represents the number of publications, with darker shades indicating higher publication counts. This suggests that the regions in darker red have higher research output in this field, while those in blue have relatively lower output.

**Figure 7:**
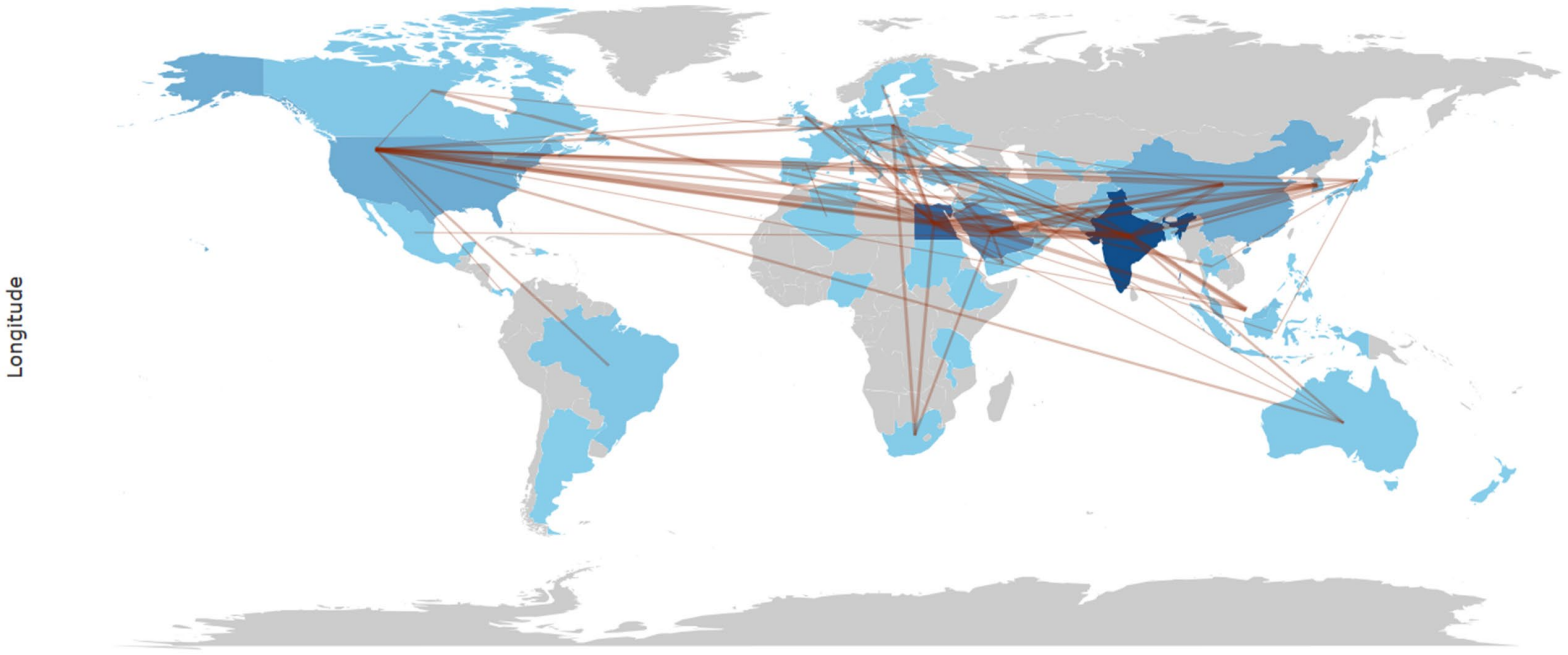
Countries collaboration network data of scientific papers reported the anticancer studies on *Aspergillus* sp. collected from Scopus database (between 1982 and 2023).

Global collaboration between countries in the field of research on *Aspergillus* species as anticancer agents is illustrated in Figure 7. The thickness of the lines may represent the strength or number of collaborations, with the countries shown in darker shades possibly having more collaborations or a central role in the research network.

### 4.3 Three field plot

**Figure 8:**
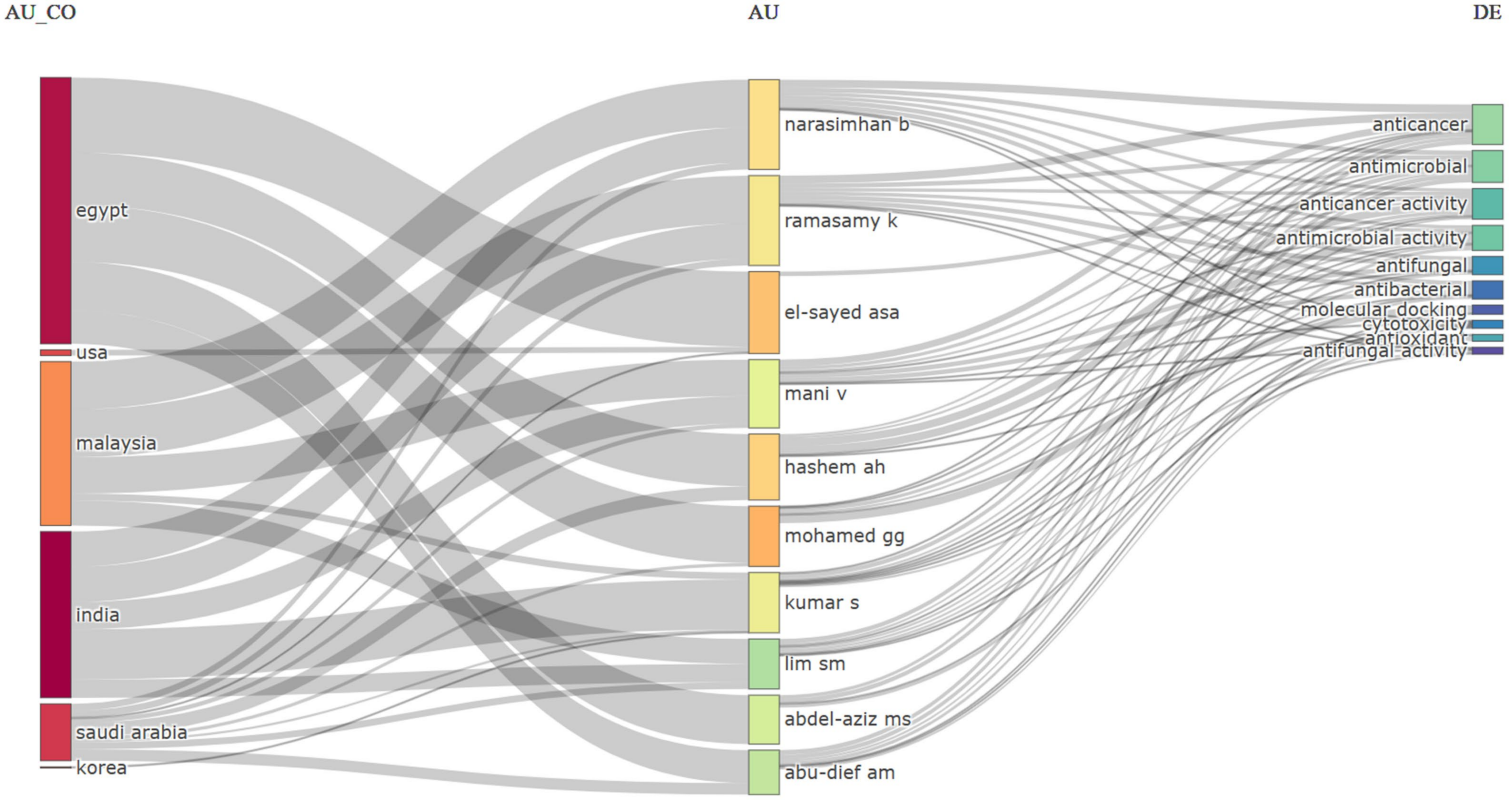
Three field plot (Country, Author name, and Keyword) of the publications on the anticancer properties of *Aspergillus* sp., analyzed from Scopus database (1982–2023).

Figure 8 depicts a Sankey diagram, a type of flow diagram where the arrow width represents the flow rate or quantity. In the given context, it illustrates the interrelation among author countries (AU_CO), individual authors (AU), and types of anti-cancer agents or activities studied in *Aspergillus* species (DE).

To interpret the diagram:

1. **AU_CO (Author Country):** This category displays countries likely representing the researchers’ origin or institutional affiliation, such as Egypt, USA, Malaysia, India, Saudi Arabia, and Korea. This indicates contributions from researchers in these nations.
2. **AU (Author):** This section presents the author’s initials and last names, likely of researchers who have published studies on *Aspergillus* species and their potential as sources of anti-cancer agents.
3. **DE (Descriptors):** This category lists various activities or properties researched in *Aspergillus* species, such as anti-cancer, antimicrobial, antifungal, and antioxidant activities.

Lines connecting countries to authors and authors to descriptors indicate which authors from which countries have contributed to researching specific aspects of *Aspergillus* species. For instance, an Egyptian author may have publications related to the anticancer and antimicrobial activities of *Aspergillus* species.

In the broader scientific research context, this diagram may be part of a bibliometric analysis, a method of quantitatively analyzing scientific literature. Such analyses unveil research patterns, identifying leading countries, prolific authors, and primary research focus areas. Here, the analysis centers around *Aspergillus* species’ potential as sources of anti-cancer compounds. *Aspergillus*, a fungi genus, produces various secondary metabolites, some with medicinal properties, including anti-cancer activities. This diagram underscores the global research effort exploring these properties for potential therapeutic applications.

### 4.4 Journal bibliometric analysis

Recognizing the trends in publications within a particular field holds significant importance for researchers aiming to find the most suitable platform to share their work or for those evaluating the overall advancement of the field. Utilizing bibliometric methods to conduct a thorough analysis of journals reveals valuable perspectives on the output of each journal. Such insights are indispensable for researchers deciding where to publish their research and for stakeholders keen on monitoring the progress of a specific research domain. Tables 3, 4 show the most relevant journal & their impact on this research domain.

**Table 3:** Top 10 journals with the highest publication output on the anticancer properties of *Aspergillus* sp., analyzed from Scopus database (1982–2023).

Journal name	Number of publications
Molecules	13
Applied Organometallic Chemistry	12
European Journal of Medicinal Chemistry	11
Marine Drugs	11
Medicinal Chemistry Research	11
Asian Journal of Pharmaceutical and Clinical Research	10
Egyptian Journal of Chemistry	10
Journal of Molecular Structure	10
Scientific Reports	10
Journal of Heterocyclic Chemistry	9

**Table 4:** Top 10 journals with the highest impact output in the field of anticancer properties of *Aspergillus* sp., analyzed from Scopus database (1982–2023).

Journal	H-Index	G-Index	M-Index	Total citations	Number of publication	Publication year start
European Journal of Medicinal Chemistry	11	11	0.55	825	11	2004
Applied Organometallic Chemistry	8	12	1.14	348	12	2017
Marine Drugs	8	11	0.8	372	11	2014
Molecules	8	13	1.33	173	13	2018
Bioorganic Chemistry	7	8	1.16	249	8	2018
Journal of Molecular Structure	7	10	1	194	10	2017
Medicinal Chemistry Research	7	11	0.58	191	11	2012
Scientific Reports	7	10	0.77	186	10	2015
Asian Journal of Pharmaceutical and Clinical Research	6	8	0.46	78	10	2011
Chemistry Central Journal	6	6	0.85	130	6	2017

**Figure 9:**
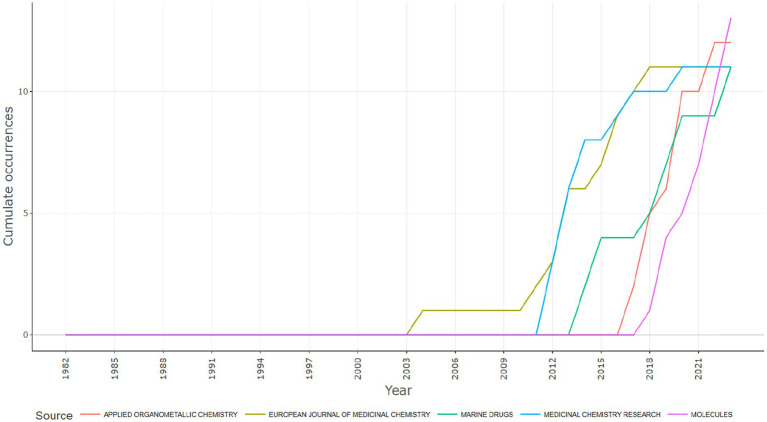
Journal production over time based on the number of publications reported the anticancer studies on *Aspergillus* sp. collected from Scopus database (Between 1982–2023).

Figure 9 illustrates the cumulative occurrences of publications across several scientific journals spanning from 1982 to 2021. The journals included in the graph are “Applied Organometallic Chemistry,” “European Journal of Medicinal Chemistry,” “Marine Drugs,” “Medicinal Chemistry Research,” and “Molecules.”

Here is a concise analysis of the graph:

- The initiation points of publication activity for each journal vary, reflecting either their respective founding years or the commencement of the time frame considered for this analysis.
- A discernible upward trend in cumulative occurrences is evident for all journals in the graph, indicating a progressive increase in research output over the years.
- Notably, “Molecules” exhibits a substantial spike in cumulative occurrences, especially prominent post the early 2000s, suggesting a significant surge in research publications within this journal during that period.
- The “European Journal of Medicinal Chemistry” also demonstrates a noticeable upswing, albeit commencing a bit earlier than “Molecules,” starting around the mid-1990s.
- In contrast, “Applied Organometallic Chemistry,” “Marine Drugs,” and “Medicinal Chemistry Research” display more modest growth patterns in comparison.

### 4.5 Bibliometric analysis of authorship

Table 5 lists authors who have contributed significantly to the field based on the number of articles published. The “Articles Fractionalized” column likely represents a fractional count of publications to account for co-authorship.

**Table 5:** Most prolific author based on publication output in the field of anticancer properties of *Aspergillus* sp., analyzed from Scopus database (1982–2023).

Authors	Articles	Articles fractionalized
Mohamed GG	15	4.66
El-Sayed ASA	14	2.95
Hashem AH	12	2.81
Narasimhan B	12	1.84
Ramasamy K	12	1.84
Kumar S	9	1.68
Mani V	9	1.36
Abdel-Aziz MS	8	1.51
Abu-Dief AM	8	1.75
Lim SM	7	1.12

This part of the analysis focuses on the impact of authors’ publications within journals, measured by indices like the H-Index, G-Index, and M-Index, alongside total citations and the start year of publication (as shown in Table 6).

**Table 6:** Top 10 authors with the highest impact output in the field of anticancer properties of *Aspergillus* sp., analyzed from Scopus database (1982–2023).

Author	H-Index	G-Index	M-Index	Total citations	Number of publications	Publication year start
Hashem AH	10	12	3.333	446	12	2021
Mohamed GG	10	15	1.429	322	15	2017
Narasimhan B	9	12	0.75	256	12	2012
Ramasamy K	9	12	0.75	256	12	2012
Abu-Dief AM	8	8	1.143	420	8	2017
El-Sayed ASA	8	14	0.8	264	14	2014
Mani V	8	9	0.667	223	9	2012
Kumar S	7	9	0.5	152	9	2010
Mahmoud WH	6	7	0.857	140	7	2017
Salem SS	6	6	2	350	6	2021

**Figure 10:**
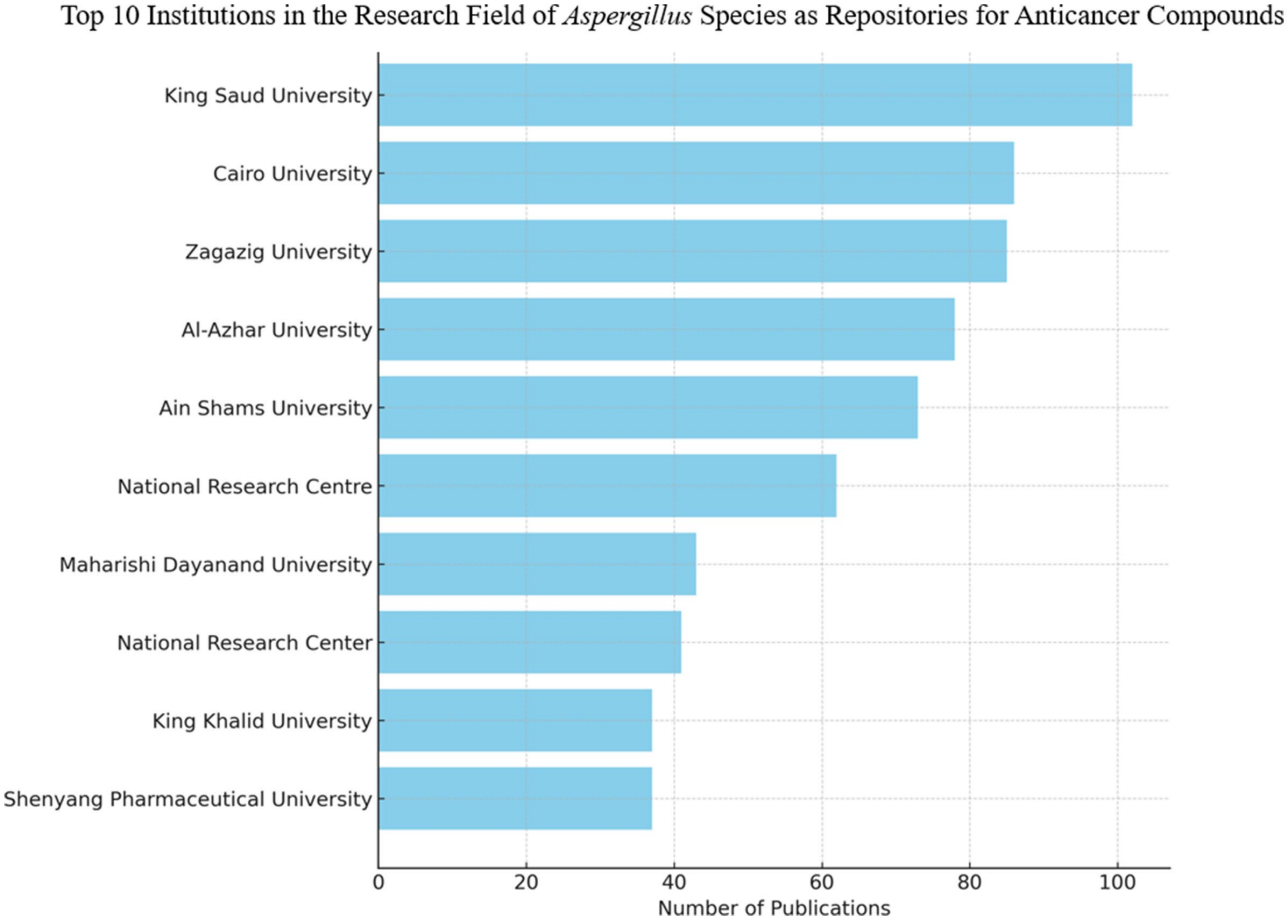
Top 10 institutions with the highest publication output on the anticancer properties of *Aspergillus* sp., Analyzed from Scopus database (1982–2023).

In this examination, the H-Index serves as a metric assessing an author’s overall impact and productivity, while the G-Index enhances the H-Index by assigning greater significance to highly cited articles. The M-Index is calculated by dividing the H-Index by the number of years since the author’s initial publication. Total Citations represent the cumulative number of citations received by the author’s works, and the Number of Publications indicates the number of articles published by the author. The Publication Year Start indicates the commencement of the author’s contributions to the field based on the dataset. This information proves essential for comprehending the influence and productivity of individual researchers within their respective domains. Figure 10 presents a ranking of the top 10 institutions in the field of *Aspergillus* species research for anticancer agents.

### 4.6 Author production analysis

**Figure 11:**
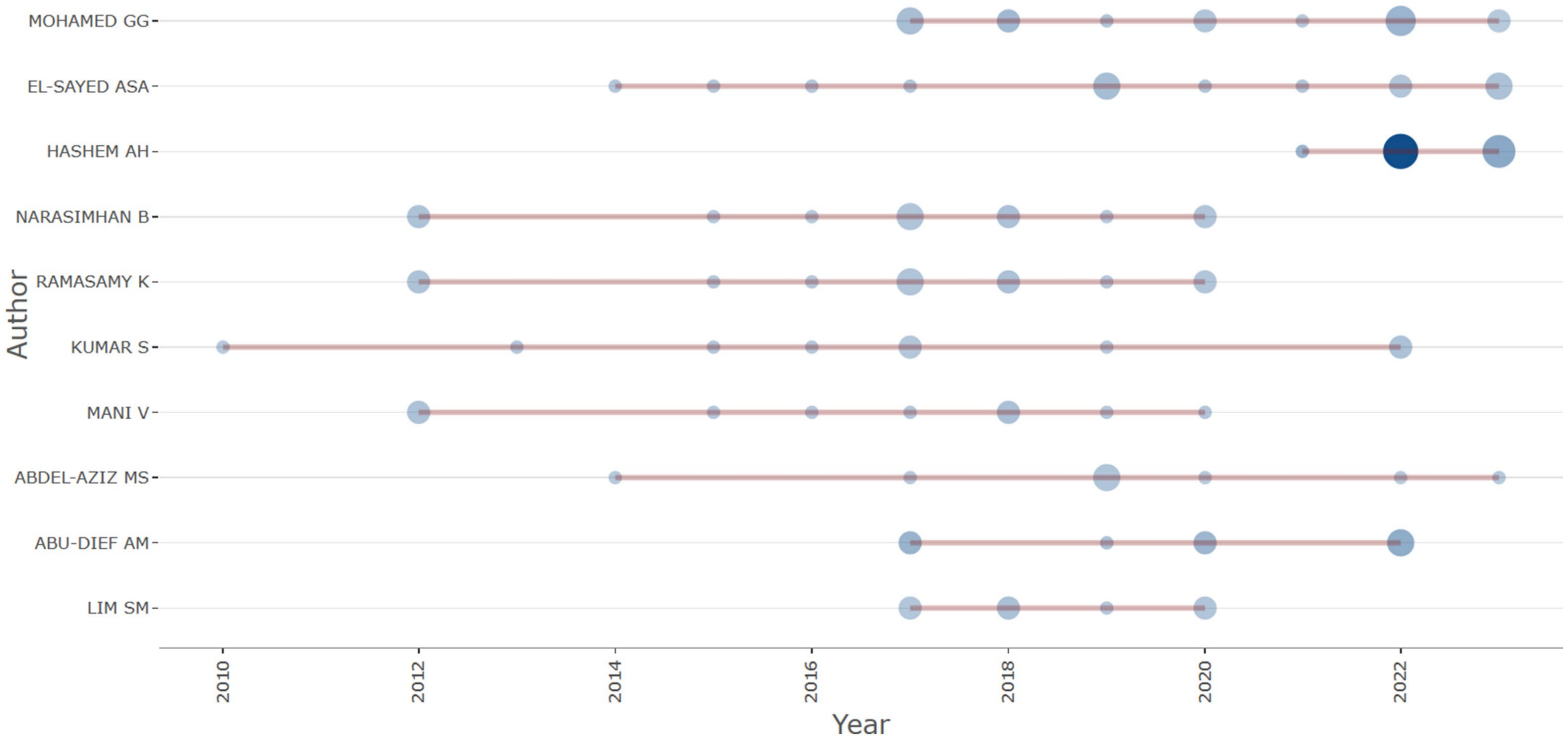
Author scientific production of publications overtime on the anticancer properties of *Aspergillus* sp., Analyzed from Scopus database (1982–2023).

Figure 11 depicts a bubble timeline chart that visually represents the publication output of various authors engaged in *Aspergillus* research for anticancer agents over time. An analysis of the elements in the graph reveals:

- **Y-axis (Author):** The vertical axis enumerates the names of authors actively involved in this research field.
- **X-axis (Year):** The horizontal axis spans from 2010 to 2022, representing the timeline.
- **Bubbles:** Each bubble corresponds to a publication by an author in a specific year. Bubble size likely correlates with the quantity or significance of publications, potentially reflecting factors like the number of papers or the impact factor of the respective journals.

From the chart, several observations can be drawn:

1. **Multiple Authors:** Notable researchers such as Mohamed GG, El-Sayed ASA, and others are listed, signifying key contributors in the field.
2. **Distribution Patterns:** The arrangement of bubbles along the timeline for each author indicates their publication frequency and potentially reflects their impact in the field.
3. **Publication Patterns:** Some authors exhibit a consistent spread of publications over the years, suggesting a sustained research effort. In contrast, others may display periods of heightened activity followed by intervals of reduced or no activity.
4. **Recent Activity:** The author with the largest bubble in the most recent year may suggest a noteworthy publication or an increase in research activity during that period.

This visual representation proves valuable for swiftly identifying leading authors, comprehending their publication trends, and analyzing shifts in research activity over time. It aids researchers and policymakers in recognizing key contributors, potential collaborators, and the field’s evolution in terms of research output.

### 4.7 Keyword analysis

In our study, we have conducted an in-depth analysis of the most frequently used keywords, which are pivotal for understanding the current trends in specific research domains. Table 7 lists the top 15 most frequently occurring author’s keywords based on total linkage strength.

**Table 7:** Top 15 most frequently occurring keywords among the publications of the anticancer properties of *Aspergillus* sp., analyzed from Scopus database (1982–2023).

S. No	Keyword	Occurrence	Total linkage strength
1	Anticancer	166	320
2	Antimicrobial	127	257
3	Antioxidant	74	194
4	Anticancer activity	113	175
5	Antibacterial	114	156
6	Antifungal	63	148
7	Cytotoxicity	65	147
8	Molecular Docking	64	121
9	Endophytic fungi	40	82
10	Apoptosis	27	57
11	Cytotoxic	26	56
12	Silver nanoparticles	30	51
13	Green synthesis	19	47
14	*Aspergillus niger*	16	43
15	*Aspergillus terreus*	13	43

**Figure 12:**
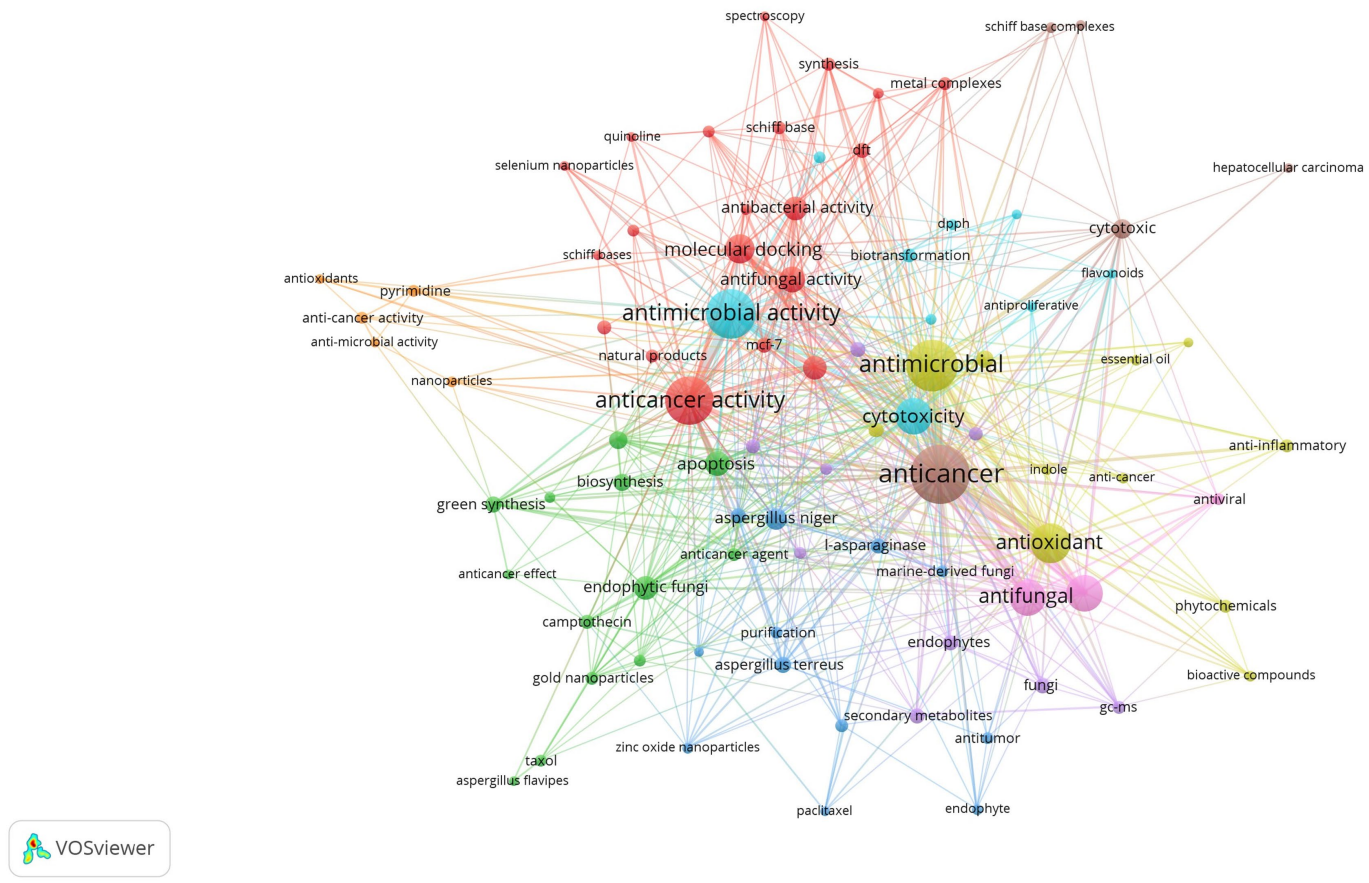
Network visualization of most occurring keyword among the publications of the anticancer properties of *Aspergillus* sp., analyzed from Scopus database (1982–2023) based on total linkage strength using Vosviewer.

Vosviewer is employed for visualizing a network of keyword occurrences based on total linkage strength (as shown in Figure 12). Out of the initial pool of 2,263 keywords, a specific criterion was applied, selecting keywords with a minimum of 5 occurrences. Eighty-seven keywords met this criteria and were utilized for the network visualization, as illustrated in the figure.

### 4.8 Word cloud

**Figure 13:**
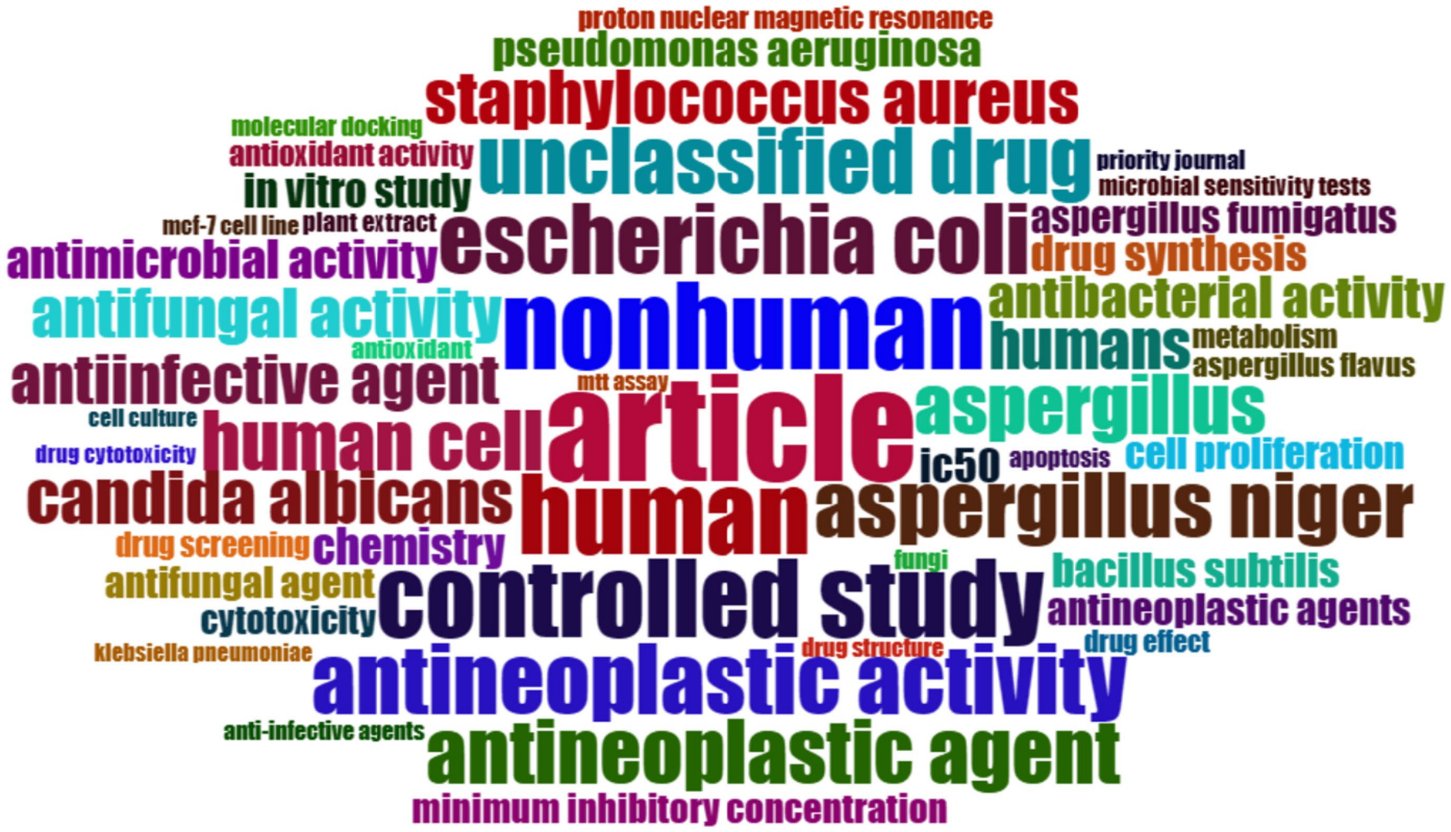
Word cloud of most frequent occurring keyword among the publications of the anticancer properties of *Aspergillus* sp., analyzed from Scopus database (1982–2023) and prepared using R-Studio.

Figure 13 is a word cloud, offering a visual representation of text data where the size of each word indicates its frequency or importance in the context of research on *Aspergillus* species as potential anti-cancer agents. A detailed analysis based on the words in the cloud is as follows:

1. **“Article” and “Study”:** Prominent in the cloud, these words suggest that the word cloud is derived from a body of literature, likely scholarly articles focused on studies in the field.
2. **“*Aspergillus,” “Aspergillus niger,” and “Candida albicans*”:** These words denote various species of fungi, with *Aspergillus niger* known for its biotechnological applications, including the production of anti-cancer compounds.
3. **“Antineoplastic Agent” and “Anticancer”:** Frequent inclusion of these terms indicates a primary focus on substances inhibiting or preventing tumor growth, underscoring the therapeutic aspect of the research.
4. **“Antimicrobial,” “Antibacterial,” and “Antifungal Activity”:** These terms imply a broad spectrum of bioactivity under investigation, extending beyond anti-cancer properties to antimicrobial effects.
5. **“Nonhuman,” “Human,” “Controlled Study”:** The presence of these terms suggests a comprehensive approach covering both nonhuman (*in vitro*) and human (clinical) research, possibly including controlled trials for rigorous scientific inquiry.
6. **“IC50,” “Cytotoxicity,” “Cell Proliferation”:** These terms, rooted in pharmacology and cellular biology, signify critical measures in cancer research. IC50 represents the concentration needed for 50% inhibition *in vitro*, while cytotoxicity and cell proliferation are pivotal in understanding the effects on cancer cells.
7. **“*Staphylococcus aureus,” “Escherichia coli,” “Pseudomonas aeruginosa*”:** Reference to these bacterial species commonly used in antimicrobial testing suggests a benchmark for evaluating potential anti-cancer drugs with antimicrobial properties.
8. **“***In Vitro* **Study,” “Cell Line,” “MTT Assay”:** These terms point towards laboratory methods and tools employed to study the impact of *Aspergillus*-derived compounds on cancer cells.

In essence, this word cloud visually summarizes the key concepts and methodologies prevalent in the research literature on *Aspergillus* species as sources of anti-cancer agents. It portrays a multidisciplinary approach, encompassing microbiology, pharmacology, molecular biology, and more, highlighting the diverse strategies employed to explore the therapeutic potential of fungal metabolites.

### 4.9 Word frequency over time

**Figure 14:**
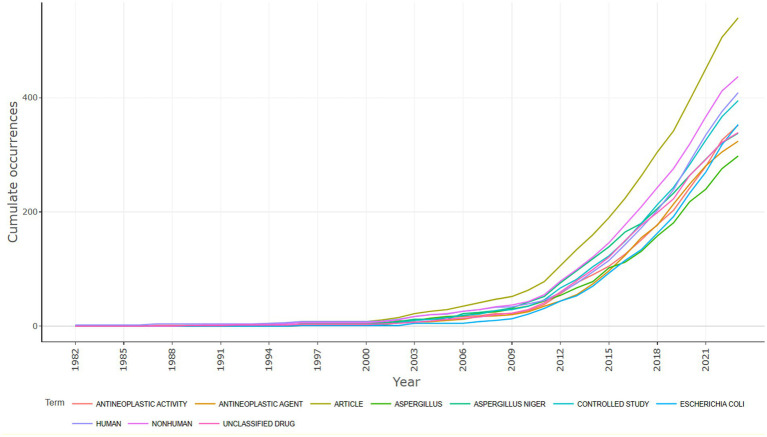
Word frequency over time among the publications of the anticancer properties of *Aspergillus* sp., analyzed from Scopus database (1982–2023) and prepared using R-Studio.

Figure 14 depicts a cumulative line graph that traces the frequency of specific terms over time in the research domain focused on *Aspergillus* as a potential source of anti-cancer agents. The X-axis, spanning from 1982 to 2021, represents the timeline for data collection, while the Y-axis quantifies cumulative occurrences, illustrating an increasing count over the years.

Each line on the graph corresponds to distinct terms, including categories like “Antineoplastic Activity,” “Antineoplastic Agent,” “Article,” “*Aspergillus*,” “*Aspergillus niger*,” “Controlled Study,” “*E. coli*,” “Human,” “Nonhuman,” and “Unclassified Drug.”

Key observations reveal a general upward trend for all terms, particularly accelerating around the early 2000s, indicative of rising interest and an expanding body of research in these realms. The growth in both “Human” and “Nonhuman” terms suggests research conducted in clinical and preclinical settings.

Notably, terms related to anti-cancer efforts, such as “Antineoplastic Activity” and “Antineoplastic Agent,” exhibit a significant increase, signaling an intensified exploration of *Aspergillus*-derived compounds for cancer treatment and prevention. The ascending lines for “*Aspergillus*” and “*Aspergillus niger*” align with increased attention towards these fungi concerning their potential anti-cancer properties.

The sustained rise in the term “Controlled Study” indicates a growing application of rigorous scientific methodologies in this field over time. The inclusion of “*E. coli*,” although not directly tied to anti-cancer research, implies potential comparative studies on the antimicrobial properties of *Aspergillus*-derived compounds, given *E. coli’s* common use as a model organism.

Lastly, the presence of “Unclassified Drug” suggests an exploration of novel or not yet fully categorized compounds derived from *Aspergillus,* emphasizing the ongoing investigation into new therapeutic possibilities.

In summary, the graph visually summarizes the increasing prominence of specific research topics and methodologies over nearly four decades within the *Aspergillus* research field. It effectively conveys the cumulative interest and expanding research efforts into *Aspergillus* species and their potential applications in the realm of cancer treatment.

### 4.10 Thematic mapping

**Figure 15:**
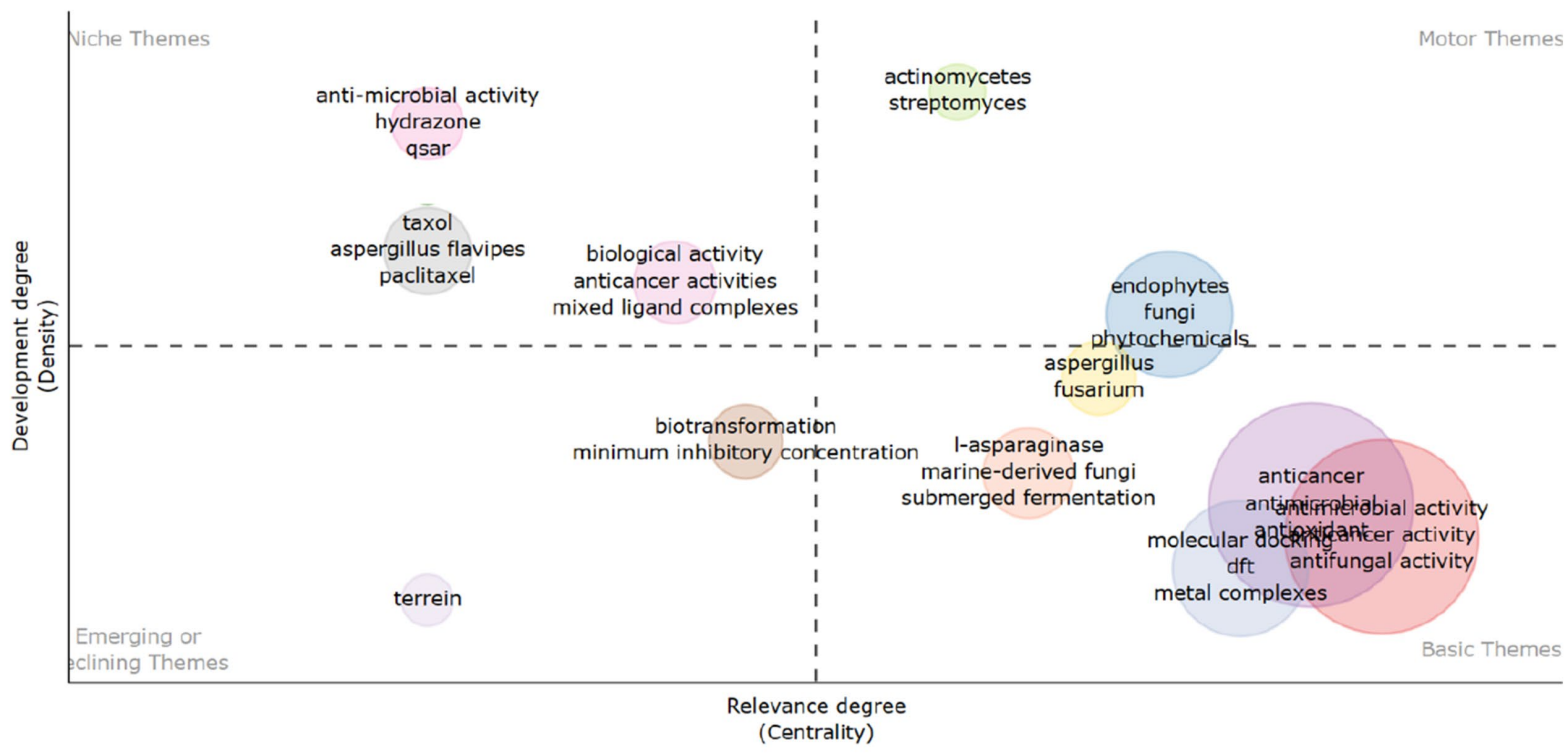
Thematic mapping of the publications on the anticancer properties of *Aspergillus* sp., analyzed from Scopus database (1982–2023).

Figure 15 is commonly known as a “strategic quadrant” or “strategy canvas.” It finds application in diverse fields, including scientific research, to visually map themes or topics based on two axes: “Development Degree” and “Relevance Degree (Centrality).” In this context, these axes denote the maturity of the research and its centrality to the field, respectively.

Regarding *Aspergillus* species and their potential as reservoirs for anti-cancer agents, the figure illustrates the positioning of *Aspergillus*-related themes within various quadrants:

1. Basic Themes: These encompass well-established and central themes, denoted by terms such as “anticancer,” “antimicrobial activity,” “anti-inflammatory,” “antioxidant,” “antifungal activity,” and “metal complexes.” This positioning suggests widespread recognition of the role played by *Aspergillus* species in producing compounds with these activities, underscoring their fundamental importance in the field of study.
2. Niche Themes: Representing less developed and less central areas of research, these include “anti-microbial activity,” “hydrazone,” and “QSAR.” Such themes may signify emerging or specialized niches within the broader study of *Aspergillus.*
3. Motor Themes: These are highly developed and central themes, such as *“Actinomycetes”* and “*Streptomyces*.” While not directly related to *Aspergillus,* these terms may indicate analogous or competing sources of anti-cancer agents in microbial bioprospecting.
4. Emerging or Declining Themes: Encompassing themes that lack both development and centrality, examples include “terrein.” These may signify either emerging areas of research or subjects losing focus.

The positioning of terms like *“Aspergillus,”* “*Fusarium,*” “phytochemicals,” “endophytes,” “fungi,” “marine-derived fungi,” and “*Aspergillus*” across the Motor and Basic Themes quadrants indicate a prominent and central role for *Aspergillus* species in producing biologically active compounds. Specifically, in the context of anti-cancer activity, it suggests that *Aspergillus* species are highly regarded as a source for discovering and developing new anti-cancer agents, given their involvement in generating diverse secondary metabolites with potential therapeutic effects.

### 4.11 Multi-correspondence analysis

**Figure 16:**
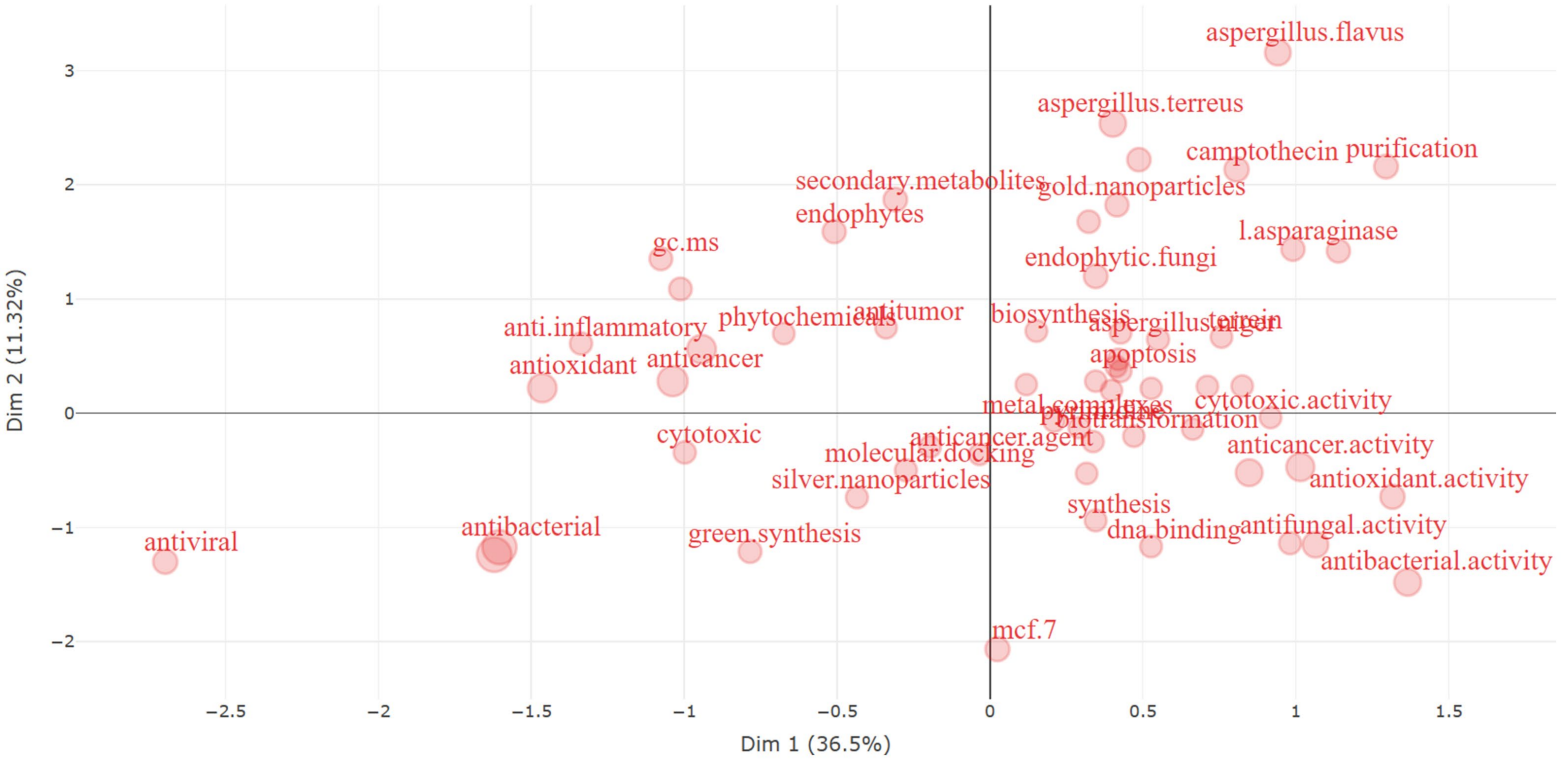
Multi-correspondence analysis plot of the publications on the anticancer properties of *Aspergillus* sp., analyzed from Scopus database (1982–2023).

The following Figure 16 is a biplot derived from a multi-correspondence analysis (MCA) analysis or a similar dimensionality reduction technique, which is often used in bioinformatics and data analysis to visualize the similarity of variables in a lower-dimensional space.

The image shows that the horizontal axis (Dim 1) captures 36.5% of the variance in the dataset, while the vertical axis (Dim 2) captures 11.23%. This indicates that Dim 1 is the most influential factor in this dataset, but together, these dimensions account for less than half of the total variance, suggesting that the underlying data is quite complex.

The terms plotted are likely keywords or terms extracted from scientific literature related to *Aspergillus* species and their potential biomedical applications. Here are some insights based on the grouping of terms:

1. **Anticancer Potential**: The terms “Anticancer,” “Antitumor,” “Cytotoxic,” and “Apoptosis” are clustered, suggesting a strong association between *Aspergillus* species and research into their anticancer properties. Apoptosis is the process of programmed cell death, and agents that can induce apoptosis are often explored for cancer therapy.
2. **Antimicrobial Activity**: The words “Antibacterial,” “Antifungal,” and “Antiviral” form another cluster, indicating that *Aspergillus* species may also be studied for their potential to fight various infections.
3. **Synthesis Methods**: “Green synthesis” is close to “Silver Nanoparticles” and “Gold Nanoparticles,” which might indicate investigations into environmentally friendly methods of synthesizing nanoparticles using *Aspergillus* species.
4. **Bioinformatics Tools**: The term “Molecular docking” is a bioinformatics method used to predict how a small molecule, such as a drug, interacts with a protein, suggesting that this technique is being used to study the interaction between *Aspergillus*-derived compounds and their potential targets in cancer cells.
5. **Specific Species and Compounds**: Specific *Aspergillus* species like “*Aspergillus terreus*” and “*Aspergillus flavus*” are mentioned alongside compounds like “Camptothecin” which is known for its anti-cancer properties, implying that these species may produce or be used to produce such compounds.
6. **Secondary Metabolites**: “Secondary Metabolites,” “Endophytes,” and “Phytochemicals” are also prominent, which points to the interest in the complex chemicals produced by *Aspergillus* that may have medicinal properties.
7. **Cell Lines**: The term “MCF 7” refers to a breast cancer cell line that is often used in cancer research, suggesting that studies are evaluating *Aspergillus* compounds against this particular type of cancer.

In summary, the visualization suggests a rich body of research focused on the bioactive potential of *Aspergillus* species, spanning anticancer activity, antimicrobial properties, and green synthesis methods. The analysis of such a biplot helps researchers identify trends, generate hypotheses, and select the most promising directions for further study.

## 5 Challenges and future perspective

### 5.1 Challenges

- The complexity of *Aspergillus* Metabolites: Unravelling the intricate biochemical pathways and the wide array of secondary metabolites generated by *Aspergillus* species (Siddhardha et al., 2010).
- Drug Resistance: Confronting the evolving resistance to current anti-cancer agents and exploring how compounds derived from *Aspergillus* may offer solutions to overcome this challenge (Sajna et al., 2020).
- Safety and Toxicity: Assessing the safety profile and potential toxicity of novel compounds sourced from *Aspergillus* species (Barba-Ostria et al., 2022).
- Scalable Production: Formulating efficient and scalable techniques for producing bioactive compounds from *Aspergillus* (Yuan et al., 2022).
- Regulatory Hurdles: Navigating the intricate regulatory landscape for the approval of new drugs derived from natural sources (Atanasov et al., 2021).

### 5.2 Future perspective

- Genomic and Metabolomic Advancements: Employing genomic and metabolomic technologies to uncover and characterize novel compounds derived from *Aspergillus* (Kalia et al., 2022).
- Targeted Drug Delivery: Examining *Aspergillus* metabolites for application in targeted drug delivery systems aimed at enhancing the effectiveness of cancer treatment (Veselov et al., 2022).
- Combination Therapies: Assessing the potential of compounds sourced from *Aspergillus* in combination therapies with existing cancer treatments (Mokhtari et al., 2017).
- Personalized Medicine: Employing *Aspergillus* compounds in personalized medicine approaches, tailoring treatments based on individual genetic profiles and specific cancer types (Goetz and Schork, 2018).
- Sustainable Bioprospecting: Advocating for sustainable and ethical bioprospecting practices in exploring *Aspergillus* species, ensuring the investigation does not harm natural ecosystems (Kuhad, 2012).

## 6 Conclusion

This research has emphasized the considerable promise of *Aspergillus* species in the development of anti-cancer agents, as indicated by our comprehensive bibliometric analysis. The varied bioactive compounds discovered in *Aspergillus,* including alkaloids, butenolides, terpenoids, and polyketides, exhibit significant effectiveness against diverse cancer types. The rising number of publications and international research collaborations in this realm reflects the increasing acknowledgment of natural products in cancer treatment and underscores the importance of sustaining this exploration.

Nevertheless, challenges persist in fully unlocking the potential of these compounds. There is a crucial need for a deeper understanding of their biosynthetic pathways, improved extraction and synthesis methods, and thorough clinical evaluations. This study highlights the essentiality of an integrative approach that combines traditional knowledge with modern scientific techniques to harness these natural resources more efficiently. The encouraging outcomes from research on *Aspergillus* species provide the impetus for ongoing and collaborative endeavors in the domain of natural product drug discovery, presenting new possibilities for safer and more efficacious cancer therapies.

## Author contributions

HJ: Conceptualization, Writing – original draft. SG: Conceptualization, Writing – original draft. PK: Conceptualization, Writing – review & editing. AK: Conceptualization, Writing – review & editing. AS: Supervision, Writing – review & editing. GK: Conceptualization, Supervision, Writing – review & editing.
